# Interaction Network Characterization of Infectious Bronchitis Virus Nsp2 with Host Proteins

**DOI:** 10.3390/vetsci11110531

**Published:** 2024-10-31

**Authors:** Mengmeng Wang, Zongyi Bo, Chengcheng Zhang, Mengjiao Guo, Yantao Wu, Xiaorong Zhang

**Affiliations:** 1 Jiangsu Co-Innovation Center for the Prevention and Control of Important Animal Infectious Disease and Zoonoses, College of Veterinary Medicine, Yangzhou University, Yangzhou 225009, China; dx120200166@stu.yzu.edu.cn (M.W.); zybo@yzu.edu.cn (Z.B.); zcc@yzu.edu.cn (C.Z.); guomj@yzu.edu.cn (M.G.); 2 Joint International Research Laboratory of Agriculture and Agri-Product Safety, The Ministry of Education of China, Yangzhou University, Yangzhou 225009, China

**Keywords:** infectious bronchitis virus, Nsp2, yeast two-hybrid, molecular docking, protein interaction, DNAJA1

## Abstract

Infectious bronchitis (IB) is a highly contagious acute viral disease that leads to substantial economic losses in the poultry industry. Previous research conducted in our laboratory has indicated that Nsp2 may serve as a key virulence factor within the IBV genome, as evidenced by its pronounced divergence between the field strain and its attenuated counterpart. Understanding the interaction between Nsp2 and host proteins is crucial to elucidating the role of the Nsp2 protein in the pathogenesis and proliferation of IBV. Currently, much remains to be uncovered regarding the host proteins that interact with the IBV Nsp2 protein. In this study, 10 host proteins, including COX1, COX3, NFIA, ITGA1, ATP1B1, ATP1B3, ABCB1, ISCA1, DNAJA1, and IREB2, were screened to interact with IBV Nsp2 through yeast two-hybrid experiments and molecular docking simulations. Furthermore, the interaction of Nsp2 with ATP1B3, DNAJA1, and ISCA1 proteins was further validated through co-immunoprecipitation and confocal experiments. The GO, KEGG, and PPI databases revealed that the host proteins interacting with Nsp2 are primarily associated with ATPase activation, Fe-S cluster binding, ion homeostasis, and innate immune regulation. The examination of the expression levels of these Nsp2-interacting host proteins during IBV infection demonstrated the significant downregulation of COX3, COX1, ATP1B1, and ATP1B3, while NFIA, DNAJA1, and IREB2 showed significant upregulation. Moreover, our study identified that IBV enhances viral replication by upregulating DNAJA1 expression, although the underlying mechanism requires further investigation. These findings provide valuable insights into the potential role of the Nsp2 protein in the pathogenesis of IBV.

## Simple Summary

Infectious bronchitis (IB) is a highly contagious viral disease that affects poultry and leads to significant economic losses. Previous studies have demonstrated that Nsp2 functions as a potential virulence factor for infectious bronchitis virus (IBV). However, the host proteins that interact with Nsp2 and their roles in IBV pathogenesis remain largely unidentified. In this study, we identified ten host proteins that interact with IBV Nsp2 through yeast two-hybrid assays and molecular docking simulations. The intracellular interactions of Nsp2 with the proteins ATP1B3, DNAJA1, and ISCA1 were further validated using co-immunoprecipitation and confocal microscopy. Analyses incorporating Gene Ontology (GO), the Kyoto Encyclopedia of Genes and Genomes (KEGG), and the Protein-Protein Interaction (PPI) database revealed that these host proteins are involved in ATPase activation, iron-sulfur (Fe-S) cluster binding, ion homeostasis, and the regulation of innate immunity. Furthermore, we observed significant changes in the expression levels of these Nsp2-interacting host proteins during IBV infection. This study establishes a foundation for the further exploration of the role and mechanisms of Nsp2 in IBV proliferation and provides valuable insights for developing novel antiviral strategies.

## 1. Introduction

Infectious bronchitis virus (IBV) is a positive-sense, single-stranded RNA virus belonging to the γ-Coronavirus genus of the Coronaviridae family. It replicates not only in the respiratory tract but also in various tissues such as the alimentary tract, kidneys, oviduct, and testes, leading to corresponding pathological changes [1,2]. The genome of IBV is approximately 27.6 kb, comprising 5′ and 3′ untranslated regions, as well as open reading frames encoding structural and non-structural proteins. Among these, ORF1ab is cleaved by the viral proteases PL^pro^ and 3CL^pro^ to 15 non-structural proteins (Nsp2 to Nsp16) [3]. These non-structural proteins play crucial roles in regulating viral replication, modulating host infection, and evading host immune responses [4,5,6].

Research conducted in our laboratory has identified Nsp2 as the protein exhibiting the highest number of amino acid mutations during IBV attenuation, and it is speculated to play a role in the regulation of IBV replication and pathogenicity [7]. Comprising 674 amino acids, Nsp2 is the first protein translated and processed during IBV replication. While dispensable for viral replication in SARS-CoV, Nsp2 interacts with various non-structural proteins, suggesting its involvement in the regulation of viral replication [8,9]. A structural analysis of SARS-CoV-2 Nsp2 has revealed three zinc finger domains, suggesting involvement in nucleic acid binding and intracellular signaling regulation [10]. Recent studies have indicated that the SARS-CoV-2 Nsp2 promotes viral replication by inhibiting Ifnb1 mRNA translation via interaction with the GIGYF2 protein [11]. Additionally, Nsp2 has been identified as a virulence factor in PEDV infection [12]. These findings imply that the Nsp2 protein is involved in various processes including coronavirus infection, replication, and pathogenicity. The structural analysis of IBV Nsp2 suggests potential involvement in early host immune response regulation during viral infection [13,14]. Furthermore, IBV Nsp2 has been found to act as a protein kinase R (PKR) antagonist in regulating host protein translation [15]. Despite these advancements, the specific function and mechanism of action of IBV Nsp2 in viral proliferation remain unclear.

The interaction between viral proteins and host proteins plays a pivotal role in pathogen propagation. Previous studies have underscored the importance of various coronavirus Nsp2 proteins in viral infection through protein-protein interactions. Host proteins that interact with MHV Nsp2 are associated with processes such as vesicle trafficking, protein translation, and autophagy [16]. Research has revealed that the Nsp2 protein of SARS-CoV modulates mitochondrial biogenesis by interacting with PHB1 and PHB2, thereby impacting the cellular environment [17]. Similar findings have also been reported for SARS-CoV-2 Nsp2. Moreover, investigations into SARS-CoV-2 host-virus interactions have identified several host proteins, including ATP6A1, TMEM199, STAU2, and WASH, that interact with Nsp2 [18,19,20]. These studies suggest that Nsp2 is involved in various biological processes, such as the regulation of mitochondrial function, apoptosis pathways, vesicular transport, and endosome transport. Nevertheless, limited research has been conducted on the interaction between the IBV Nsp2 protein and host proteins.

In this study, 10 host proteins were screened for their interaction with IBV Nsp2 through yeast two-hybrid experiments and molecular docking simulations. The intracellular interaction between Nsp2 and ATP1B3, DNAJA1, and ISCA1 proteins was further confirmed by co-immunoprecipitation and confocal experiments. The GO, KEGG, and PPI databases revealed that these host proteins participate in various cellular processes such as ATPase activation, Fe-S cluster binding, ion homeostasis, and innate immune regulation. Further investigations demonstrated that IBV promotes viral self-replication by upregulating DNAJA1 expression. This study lays the foundation for further exploring the role and mechanism of Nsp2 in IBV proliferation, providing valuable insights for the development of novel antiviral strategies.

## 2. Materials and Methods

### 2.1. Cells, Strains, and Antibodies

CEK cells were aseptically prepared from 20-day-old specific-pathogen-free (SPF) chicken embryos. The kidneys were trypsinized at 37 °C for 30 min, and the cell suspensions were then filtered through a 100 µm mesh. Subsequently, the cells were cultured in M199 medium (M199; Gibco, New York, NY, USA) containing 5% fetal bovine serum (FBS; Gibco, New York, NY, USA). HEK293T and DF-1 cells were purchased from ATCC and cultured in Dulbecco’s modified Eagle medium (DMEM; Gibco, New York, NY, USA) supplemented with 10% FBS at 37 °C in a 5% CO_2_ incubator. The JS/2010/12 strain of IBV (GenBank accession No. PP100175) was isolated and stored in our laboratory. Mouse anti-Flag, mouse anti-Myc, and mouse anti-GAPDH antibodies were purchased from Sigma (St. Louis, MO, USA); the mouse anti-N antibody was prepared in our laboratory. Horseradish peroxidase (HRP)-conjugated goat anti-mouse IgG antibody was purchased from Bioss (Beijing, China).

### 2.2. Plasmids

All primers for amplifying the target fragment were designed with Primer 5.0, as shown in Table 1, with an annealing temperature of 50 °C. The IBV cDNA was used as the template for amplifying the Nsp2 gene. After amplification, the Nsp2 gene was cloned into pGBKT7, pEGFP-C1, and pcDNA3.1-Flag. The ATP1B3 gene (GenBank accession NM_205535.1), the DNAJA1 gene (GenBank accession NM_001012945), and the ISCA1 gene (GenBank accession NM_001271936.1) were cloned from the cDNA of CEK cells. These genes were then cloned into pDsRed and pcDNA3.1-Myc, respectively.

### 2.3. Construction and Identification of a cDNA Library from Chicken Kidney Tissue

Total RNA was extracted from the kidney tissues of 20-week-old chickens using an ultrapure RNA kit (CWBIO, Taizhou, China), following the manufacturer’s instructions. The RNA was reverse-transcribed into first-strand cDNA using SMART MMLV reverse transcriptase (Clontech, Palo Alto, CA, USA). This cDNA was then used as a template to produce double-stranded complementary DNA (ds cDNA) containing pGADT7-Rec homology arms at both ends by long-distance PCR (LD-PCR). Subsequently, a CHROMA SPIN TE-400 column was used to screen ds cDNA with molecular lengths greater than 200 bp. Purified ds cDNA and pGADT7-Rec (Clontech, Palo Alto, CA, USA) were co-transformed into Y187 cells. The cells were then resuspended in 15 mL of 0.9% (*w*/*v*) NaCl, plated on 100 plates of SD/-Leu, and then incubated at 30 °C for 3 days. Thirty-two colonies were randomly selected and identified by PCR to evaluate the insert size and library recombination rate. After chilling all plates at 4 °C for 4 h, the colonies were resuspended in a freezing medium (YPDA liquid medium containing 25% glycerol), and the library titers were subsequently calculated.

### 2.4. Yeast Two-Hybrid Screening for Nsp2 Interacting Proteins

The plasmid pGBKT7-Nsp2 was transformed into Y2H cells (Clontech, Palo Alto, CA, USA) using the PEG/LiAc method. After confirming that pGBKT7-Nsp2 did not exhibit autoactivation or toxicity, the bait strain was mixed with the cDNA library and incubated at 30 °C for 24 h. The mixed culture was then spread onto SD/-Trp/-Leu/X-α-Gal/Aba (DDO/X/A) agar plates and incubated at 30 °C for 5 days to screen for blue yeast colonies. These blue colonies were transferred onto SD/-Ade/-His/-Leu/-Trp/X-α-Gal/Aba (QDO/X/A) agar plates with higher stringency, and this process was repeated three times. Positive clones were sequenced and analyzed using NCBI BLAST (https://blast.ncbi.nlm.nih.gov/Blast.cgi, accessed on 27 April 2020) to identify the interacting host factors. Prey plasmids from the positive colonies were extracted and co-transformed with the bait plasmid pGBKT7-Nsp2 into Y2H cells to confirm the positive interactions in yeast. As positive controls, pGBKT7-53 and pGADT7-T were transformed into Y2H cells, while pGBKT7-LAM and pGADT7-T were used as negative controls.

### 2.5. Molecular Docking

The binding patterns and affinity assessment for the interactions between the Nsp2 protein and host proteins were conducted through molecular docking. The homologous structural model of the Nsp2 protein was generated using SWISS-MODEL (https://swissmodel.expasy.org/, accessed on 20 May 2024), and the three-dimensional structural models of the host proteins were obtained from the PDB database (https://www.rcsb.org/, accessed on 20 May 2024). Subsequent protein-protein docking simulations were conducted using the Z-DOCK platform (http://zdock.umassmed.edu/, accessed on 20 May 2024), with analysis of the resulting complexes performed using PDBePISA (http://www.ebi.ac.uk/msd-srv/prot_int/, accessed on 23 May 2024). The binding activity and docking effect of protein-protein interaction were assessed based on the free binding energy levels, with levels greater than −4.0 kcal/mol indicating weak or non-existent binding, levels from −7.0 to −4.0 kcal/mol indicating good binding activity, and levels less than or equal to −7.0 kcal/mol indicating strong binding activity.

### 2.6. Confocal Laser Scanning Microscopy Assay

DF-1 cells were seeded in 24-well plates with coverslips. Upon reaching 80% confluence, pDsRed-ATP1B3, pDsRed-DNAJA1, or pDsRed-ISCA1 were co-transfected with pEGFP-Nsp2. After 36 h, the cells were fixed with 4% paraformaldehyde and stained with 4′, 6-diamidino-2-phenylindole (DAPI; Sigma, St. Louis, MO, USA) for nuclei visualization. The fluorescence signal was detected using a laser confocal microscope (LSM510 META; Zeiss, Oberkochen, Batenfuburg, Germany).

### 2.7. Co-Immunoprecipitation (Co-IP)

HEK293T cells were co-transfected with pcDNA3.1-Nsp2-Flag and recombinant plasmids expressing ATP1B3, DNAJA1, or ISCA1. After 36 h, the cells were lysed using RIPA buffer (Beyotime, Nanjing, China) and centrifuged at 12,000 rpm for 10 min at 4 °C, with the supernatant being collected. The supernatants were then incubated with magnetic beads (Beyotime, Nanjing, China) adsorbed with Flag, Myc, or IgG antibodies overnight at 4 °C. The beads were then washed three times with TBST buffer, mixed with SDS-PAGE loading buffer (Beyotime, Nanjing, China), and boiled for 10 min. The proteins were separated via sodium dodecyl sulfate-polyacrylamide gel electrophoresis (SDS-PAGE) and then transferred to a polyvinylidene difluoride (PVDF) membrane (Pall, New York, NY, USA). The membrane was blocked in TBST containing 5% skimmed milk for 1 h at room temperature and then incubated with specific antibodies (1:2000 dilution) overnight at 4 °C. After washing with PBST three times, the membrane was incubated with an HRP-labelled secondary antibody (1:5000 dilution) for 2 h at room temperature. Finally, the protein bands were visualized using the ECL Plus kit (NCM Biotech, Suzhou, China) and the chemiluminescence imaging system (Tanon 6600, Shanghai, China).

### 2.8. Bioinformatic Analysis of Potential Protein Functions

The online database David (https://david.ncifcrf.gov/, accessed on 28 May 2024) was used to perform Gene Ontology (GO) enrichment and Kyoto Encyclopedia of Genes and Genomes (KEGG) pathway analysis. The GO term enrichment analysis included the molecular function (MF), biological process (BP), and cellular component (CC). The UniProt IDs of the candidate proteins were uploaded to the online tool STRING (https://string-db.org/, accessed on 28 May 2024) to retrieve protein-protein interaction (PPI). Subsequently, the STRING results were further analyzed and mapped using Cytoscape_v3.8.2.

### 2.9. siRNAs and Transfection

siRNA targeting DNAJA1 was synthesized by General Biol (Chuzhou, China) with the following sequences: si-DNAJA1: 5′-GGACCAAGGCCAGGACUA-3′; si-NC: 5′-UUCUCCGAACGUGUCACGGU-3′. CEK cells at 50% confluence were transfected with the siRNA using TransIntro EL Transfection Reagent (TransGen, Beijing, China). After 24 h, the cells were infected with IBV. Subsequently, the cells were harvested and analyzed for RNA detection and Western Blotting analysis at 12, 24, 36, and 48 h post-infection.

### 2.10. Quantitative Real-Time PCR (qRT-PCR)

Total RNA was extracted from the cells using the Ultrapure RNA Kit (CWBIO, Taizhou, China) and reverse-transcribed into cDNA. Subsequently, the mRNA levels of the target proteins and β-actin were quantified by RT-PCR using SYBR Green Master Mix (Vazyme, Nanjing, China), and all primers were annealed at 55 °C. β-actin was used as an internal reference to standardize the transcript and viral RNA levels. The fold change in mRNA expression was determined using the 2^−ΔΔCT^ method. Details of the primers used can be found in Table 2.

### 2.11. Statistical Analysis

Verification was performed more than three times for all experiments. The data are presented as the mean ± standard deviation (SD). GraphPad Prism 8.0.1 software was used to determine the statistical significance between the groups. * *p* < 0.05, ** *p* < 0.01, *** *p* < 0.001.

## 3. Results

### 3.1. cDNA Library Construction and Quality Assessment

**Figure 1:**
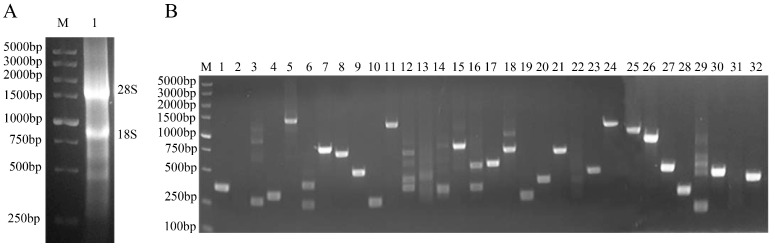
Construction and quality assessment of the cDNA library. (**A**) Electrophoretic analysis of total RNA extracted from chicken kidney tissue. Lane M represents the DL5000 DNA marker, while lane 1 shows the total RNA. (**B**) Identification of the inserted DNAs from the cDNA library. Lane M corresponds to the DL5000 DNA marker, and lanes 1−32 display 32 individual recombinant colonies that were randomly selected and amplified by PCR using universal primers of the pGADT7-Rec vector.

Total RNA was extracted from the kidney tissue of 20-week-old chickens, yielding an OD260/280 ratio of 2.04. Subsequent electrophoresis revealed two distinct RNA bands, namely, 28S and 18S, with the former displaying a higher intensity than the latter (Figure 1A), indicating intact RNA without degradation. The ds cDNA produced via LD-PCR was purified using a CHROMA SPIN TE-400 purification column. Through co-transformation of the purified ds cDNA and pGADT7-Rec into Y187 cells, a chicken kidney cDNA library was constructed with a titer of 3.9 × 10^7^ CFU/mL. Electrophoretic analysis of 32 randomly selected colonies revealed fragment sizes ranging from 200 bp to 2000 bp, demonstrating a recombination rate of 93.7% (Figure 1B).

### 3.2. Yeast Two-Hybrid Screen

**Figure 2:**
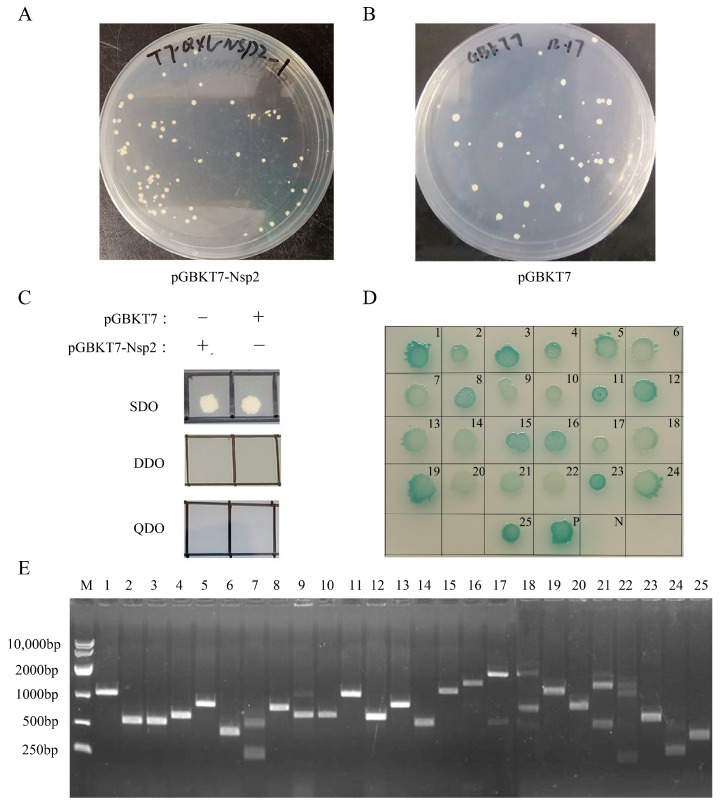
Yeast two-hybrid assay for identifying the interaction between IBV-Nsp2 and the chicken kidney cDNA library. The toxicity (**A**,**B**) and autoactivation activity (**C**) of the bait plasmid pGBKT7-Nsp2 in Y2H cells were detected. (**D**) Reversion verification test of the Nsp2 potential interaction genes. The plasmid to be verified and the pGBKT7-Nsp2 plasmid were co-transformed into Y2H yeast competent cells, cultivated on QDO/X/A plates at 30 °C for 5 days. (**E**) PCR products from positive clones of the cDNA library. Lane M was the DL10,000 DNA marker. Lanes 1−25 are 25 host proteins interacting with the IBV Nsp2 protein in the following order: ITGA1, DNAJA1, ATP1B1, ATP1B3, NFIA, RPL12, RARRES2, ND1, PCNA, LEO1, COX1, COX3, AKIP1, THEM4, PECAM1, ABCB1, COL4A4, SORBS2, IREB2, SELENBP1, NET1, LOXL1, PSMB1, ISCA1, and FAM96B.

The potential autoactivation and toxicity of the bait vector were evaluated by transforming pGBKT7-Nsp2 and pGBKT7 into Y2H cells. The size similarity of pGBKT7-Nsp2 and pGBKT7 on SD/-Trp (SDO) plates indicated the non-toxic nature of the bait plasmid (Figure 2A,B). The observation of white plaques on the SDO plate and the absence of plaques on the DDO and QDO plates suggested the absence of autoactivation by the Nsp2 protein (Figure 2C). The subsequent incubation of pGBKT7-Nsp2 with a cDNA library at 30 °C led to the identification of 92 primary positive clones following screening in selective media. Sequencing and alignment using BLAST on the NCBI website revealed that eight of the positive clones represented an unknown protein, while the remaining clones contained 25 host factors that interact with Nsp2 (Table S1). Reversion verification on QDO/X/A auxotrophic plates confirmed interaction signals with Nsp2 for all 25 proteins (Figure 2D). PCR analysis of positive clones indicated that the screened cDNA lengths from the library mainly ranged between 200 bp and 2000 bp (Figure 2E).

### 3.3. Docking Analysis and Prediction of the Interaction Between Nsp2 and Host Proteins

**Figure 3:**
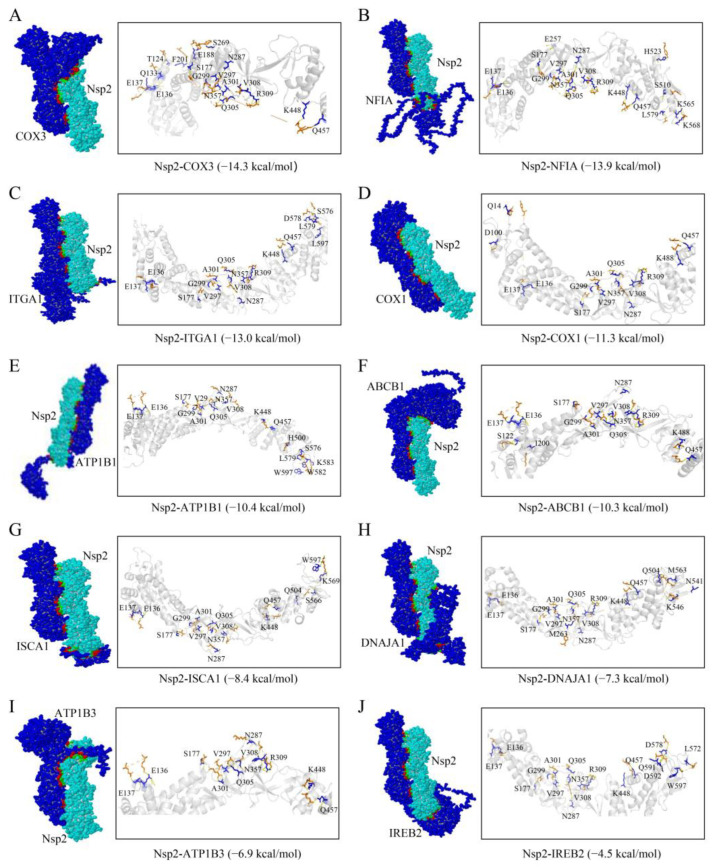
Diagram of the binding pattern of the Nsp2 protein to the host proteins (**A**) COX3, (**B**) NFIA, (**C**) ITGA1, (**D**) COX1, (**E**) ATP1B1, (**F**) ABCB1, (**G**) ISCA1, (**H**) DNAJA1, (**I**) ATP1B3, and (**J**) IREB2. The interaction residues of the Nsp2 protein with the host proteins are shown as blue sticks, while the host proteins are denoted by orange sticks.

Molecular docking simulations were conducted in this study to screen host proteins interacting with the Nsp2 protein. Nsp2 was docked individually with 25 host proteins using the Z-DOCK platform, which uses a Fast Fourier Transform algorithm to predict the structures of protein-protein complexes. The docking process scores these complexes based on a combination of shape complementarity, electrostatics, and statistical potential terms, where a higher score indicates a better binding affinity. Models with the highest docking scores were subsequently submitted to the PDBePISA server to evaluate protein-protein interactions such as binding energy and amino acid residue interactions. Binding energy was used as an indicator to evaluate the binding affinity; the more stable the protein-protein binding, the lower the binding energy of both. When the free binding energy is greater than −4.0 kcal/mol, the binding activity between the two proteins is usually considered to be weak or non-existent. The findings identified 10 host proteins exhibiting good binding affinity with Nsp2 (Figure 3). The calculated free binding energies between Nsp2 and the host proteins were as follows: cytochrome c oxidase subunit III (COX3, −14.3 kcal/mol), nuclear factor I A (NFIA, −13.9 kcal/mol), integrin subunit alpha 1 (ITGA1, −13.0 kcal/mol), cytochrome c oxidase subunit I (COX1, −11.3 kcal/mol), ATPase Na^+^/K^+^ transporting subunit beta 1 (ATP1B1, −10.4 kcal/mol), ATP binding cassette subfamily B member 1 (ABCB1, −10.3 kcal/mol), iron-sulfur cluster assembly 1 (ISCA1, −8.4 kcal/mol), DnaJ heat shock protein family member A1 (DNAJA1, −7.3 kcal/mol), ATPase Na^+^/K^+^ transporting subunit beta 3 (ATP1B3, −6.9 kcal/mol), and iron-responsive element binding protein 2 (IREB2, −4.5 kcal/mol). The potential functions of these host proteins are outlined in Table 3.

### 3.4. Nsp2 Interacts with ATP1B3/DNAJA1/ISCA1 in Cells

**Figure 4:**
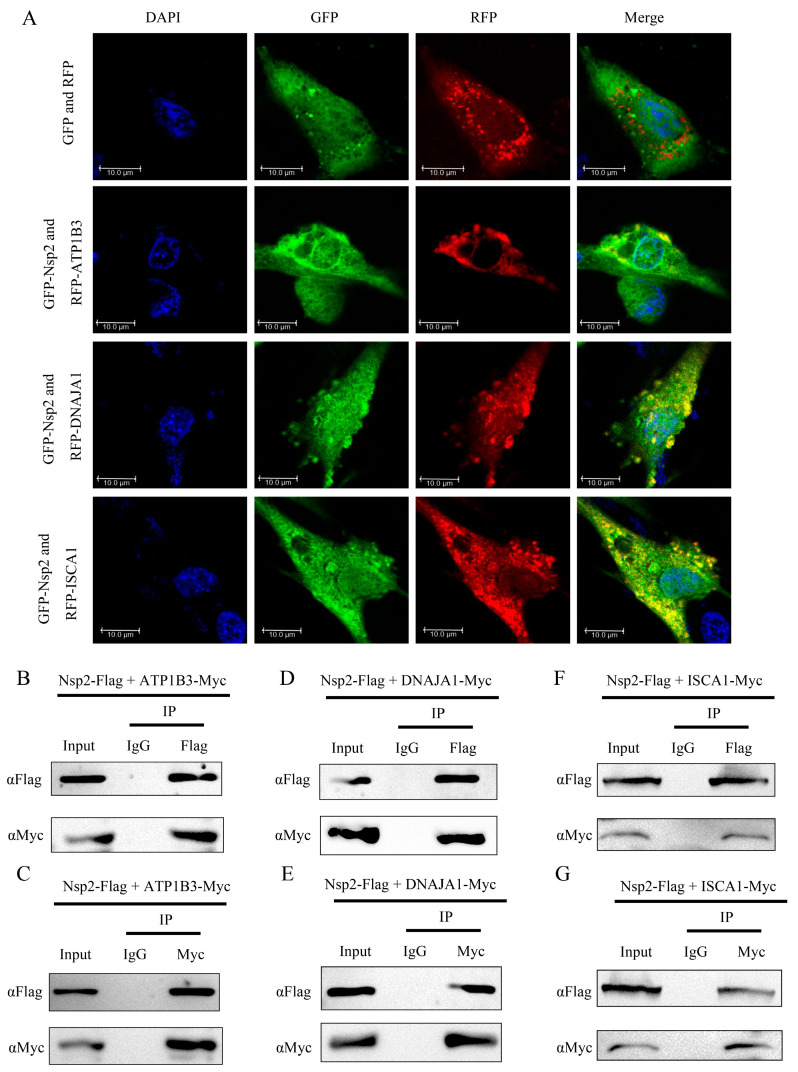
Identification of the interaction between Nsp2 and the host proteins ATP1B3, DNAJA1, and ISCA1. (**A**). Colocalization analysis between Nsp2 and the host proteins ATP1B3, DNAJA1, and ISCA1. DF-1 cells were cotransfected with pEGFP-Nsp2 and pDsRed-tagged ATP1B3, DNAJA1, or ISCA1 for 36 h, and the nuclei were stained with DAPI before observation using confocal microscopy. (**B**–**G**) Co-IP analysis to identify Nsp2 interaction with host proteins. HEK293T cells were co-transfected with pcDNA3.1-Nsp2-Flag and Myc-tagged ATP1B3, DNAJA1, or ISCA1. After 36 h, cellular protein samples were collected and separately incubated with anti-Flag magnetic Beads, anti-Myc magnetic Beads, and IgG magnetic beads. The eluted proteins were then separated by SDS-PAGE and analyzed by Western Blotting using specific primary and secondary antibodies.

In this study, the interaction between Nsp2 and selected host proteins was validated through subcellular colocalization and Co-IP experiments. DF-1 cells were co-transfected with pEGFP-Nsp2 and pDsRed-tagged ATP1B3, DNAJA1, or ISCA1, followed by observation using laser confocal microscopy. The results demonstrated the colocalization of Nsp2 with ATP1B3, DNAJA1, and ISCA1, manifesting as yellow fluorescence with a dot-like distribution (Figure 4A). Additionally, in HEK293T cells, Co-IP experiments were performed by co-transfecting Nsp2-Flag with Myc-tagged ATP1B3, DNAJA1, or ISCA1. The outcomes demonstrated the efficient capture of the host proteins by Nsp2 and vice versa, indicating an intracellular interaction between Nsp2 and ATP1B3/DNAJA1/ISCA1 (Figure 4B−G).

### 3.5. GO and KEGG Pathway Analysis of Nsp2-Interacting Proteins

**Figure 5:**
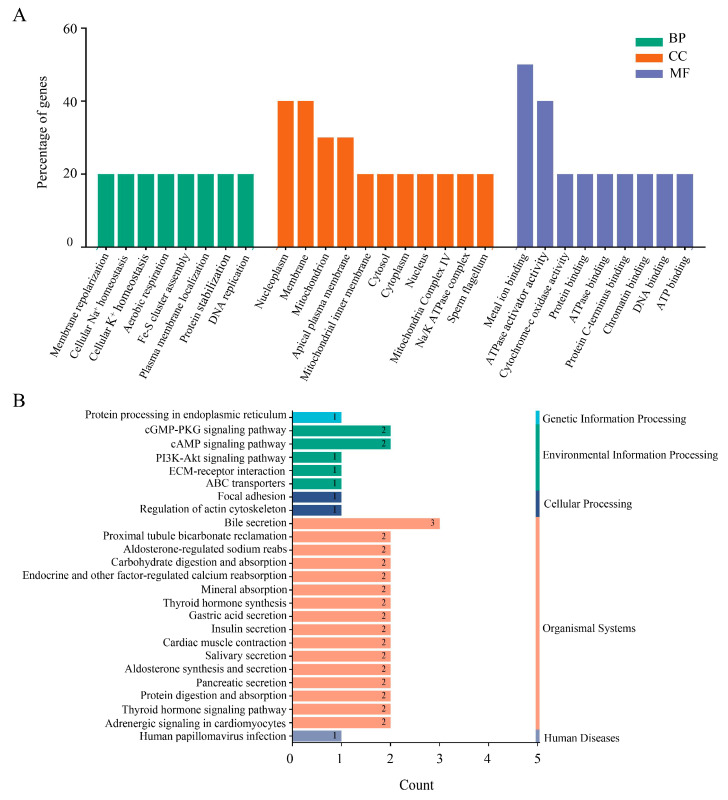
GO and KEGG pathway analysis of host proteins interacting with Nsp2. (**A**) GO enrichment analysis. BP, biological process; CC, cellular component; MF, molecular function. The horizontal axis represents GO functional categories and the vertical axis shows gene percentages. (**B**) KEGG pathway enrichment analysis. The horizontal axis represents the number of genes, and the vertical axis represents the pathway categories.

This study utilized the DAVID database to conduct GO and KEGG pathway analyses in order to investigate the functional roles of Nsp2-interacting host proteins. The results revealed that these proteins participate in various biological processes, including membrane repolarization, cellular ion homeostasis, aerobic respiration, Fe-S cluster assembly, plasma membrane localization, protein stabilization, and DNA replication. Furthermore, the proteins were predominantly located in the nucleoplasm, membrane, mitochondria, apical plasma membrane, cytoplasm, and protein complexes. They displayed various molecular functions, such as metal ion binding, ATPase activator activity, ATP binding, DNA binding, chromatin binding, and Cytochrome-c oxidase activity (Figure 5A). The analysis of the KEGG database indicated that these candidate proteins are involved in modulating the PI3K-Akt signaling pathway, cGMP-PKG signaling pathway, and cAMP signaling pathway. Moreover, these proteins are likely to be significant contributors to processes such as endocrinology, protein processing, and receptor binding (Figure 5B).

### 3.6. Construction of the Nsp2-Host Protein Interaction Network

**Figure 6:**
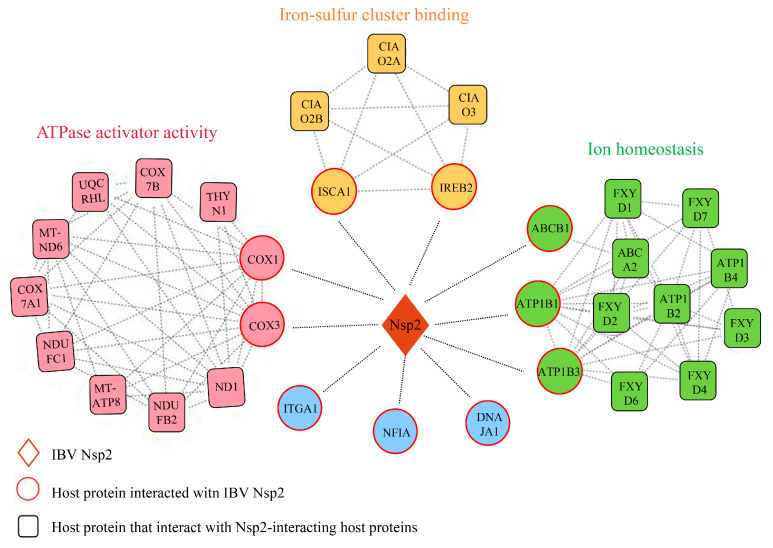
The network of interactions between IBV Nsp2 and host proteins. The protein-protein interaction network was built using the STRING database and Cytoscape. Within this network, diamonds represent Nsp2 proteins, circles depict Nsp2 candidate interacting host proteins identified through Y2H experiments, and squares illustrate other host proteins interacting with the candidate host proteins. Protein interactions are illustrated by straight lines connecting nodes.

The protein-protein interaction (PPI) network provides comprehensive insight into the interactions among various proteins. In this study, we predicted the interaction networks involving Nsp2 and candidate host proteins, along with other host proteins interacting with these candidates. Our analysis unveiled 94 edges connecting 32 host proteins in the network, which formed three distinct clusters (Figure 6). The first cluster, consisting of 11 nodes and 40 edges, was associated with ATPase activation, where COX1 and COX3 were identified as host proteins interacting with Nsp2. The second cluster, comprising 5 nodes and 10 edges, was linked to iron-sulfur cluster binding, including the candidate Nsp2-interacting host proteins ISCA1 and IREB2. The third cluster involved ATP1B1, ATP1B3, ABCB1, and other host proteins associated with ion homeostasis maintenance.

### 3.7. IBV Upregulates DNAJA1 to Facilitate Viral Replication

**Figure 7:**
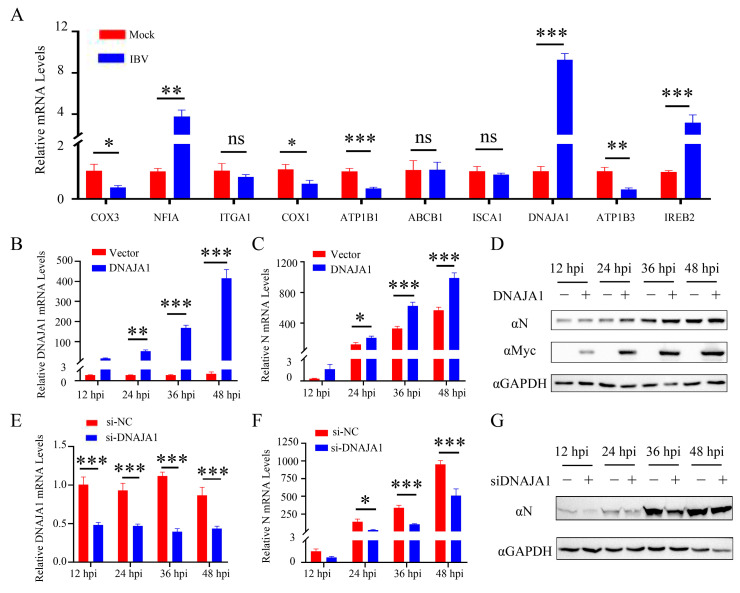
IBV infection upregulates DNAJA1 expression to promote viral replication. (**A**) The mRNA expression levels of potential host proteins interacting with Nsp2 were detected using qRT-PCR 24 h post IBV infection. Experimental data were pooled and analyzed using *t*-tests. CEK cells were transfected with pcDNA3.1-DANAJ1-Myc for 24 h and then infected with IBV; the cells were collected 12, 24, 36, and 48 h later. qRT-PCR was used to detect DNAJA1 (**B**) and IBV-N (**C**) mRNA expression levels, while Western Blotting was employed to detect the expression of indicated proteins (**D**). Additionally, CEK cells were transfected with siRNA to silence DNAJA1 for 24 h and then infected with IBV. Cells were collected 12, 24, 36, and 48 h post-inoculation. qRT-PCR was used to measure DNAJA1 (**E**) and viral N protein (**F**) mRNA expression levels, and Western Blotting was used to analyze protein expression (**G**). The data are presented as the mean ± SD. ^ns^ *p* > 0.05, * *p* < 0.05, ** *p* < 0.01, *** *p* < 0.001.

This study aimed to explore the involvement of host proteins interacting with Nsp2 in viral replication. The mRNA expression levels of these proteins were assessed in IBV-infected CEK cells using qRT-PCR. The results showed a significant decrease in the transcription levels of COX3, COX1, ATP1B1, and ATP1B3 after 24 h of IBV infection, while NFIA, DNAJA1, and IREB2 exhibited notable upregulation (Figure 7A). Subsequent investigation focused on the impact of DNAJA1 protein expression on viral replication. The study revealed that early in viral infection, the overexpression of the DNAJA1 protein boosted viral replication (Figure 7B−D), whereas interference with the DNAJA1 protein expression inhibited viral replication in CEK cells (Figure 7E−G). These results suggest that IBV can regulate the expression of host proteins interacting with Nsp2, indicating their potential significance in IBV replication.

## 4. Discussion

The interaction between viruses and hosts during infection alters the host’s innate immune and transcriptional-translational systems, impacting the viruses’ ability to manipulate host control mechanisms and promote their propagation [21]. Yeast two-hybridization is a widely employed method for efficiently screening virus-host interactions that is capable of detecting stable as well as weak or transient protein interactions [22]. Recent studies have successfully identified host proteins that interact with the Nsp1, Nsp13, and Nsp16 proteins of SARS-CoV using this approach [23,24,25]. Expanding on these findings, investigations into the impact of viral protein-host protein interactions on viral infection have been conducted. Previous research from our laboratory has suggested the potential involvement of the IBV Nsp2 protein in viral replication and proliferation, although its specific functions and mechanisms of action remain unclear [7]. In this study, we screened 25 host proteins that interact with IBV Nsp2 from a chicken kidney cDNA library by yeast two-hybrid screening (Figure 1 and Figure 2). To address potential false positives from the screening, a molecular docking simulation was performed for reassessment. Ultimately, we confirmed the interaction of Nsp2 with 10 host proteins, namely, COX1, COX3, NFIA, ITGA1, ATP1B1, ATP1B3, ABCB1, ISCA1, DNAJA1, and IREB2 (Figure 3). Furthermore, the intracellular interactions of ATP1B3, DNAJA1, and ISCA1 with Nsp2 proteins were validated through immunoprecipitation and confocal assays (Figure 4). These findings provide compelling support for the combined utility of yeast two-hybrid and molecular docking simulation in host protein screening.

The upregulation of SARS-CoV proteins has been linked to elevated mitochondrial transmembrane potential (∆Ψm), reactive oxygen species (ROS) generation, and the induction of cellular apoptosis [26]. Among the host proteins screened in this investigation, COX1, COX3, DNAJA1, ISCA1, and IREB2 are all related to mitochondrial biogenesis (Figure 5 and Figure 6). COX1 and COX3, key mitochondrial-encoded proteins critical for aerobic respiration [27], showed downregulated expression levels after IBV infection (Figure 7), consistent with previous research on SARS-CoV-2 [28]. This downregulation may disrupt electron transfer efficiency, leading to increased ROS production and ultimately resulting in cellular damage or apoptosis [29]. The cytoplasmic chaperone DNAJA1-HSP70 is known to play a crucial role in preventing NO-mediated apoptosis released by mitochondrial cytochrome C [30]. ISCA1 and IREB2 are both involved in the biogenesis of Fe-S clusters within mitochondria. ISCA1 is indispensable for the maturation of mitochondrial 4Fe-4S proteins and contributes to diverse cellular processes including mitochondrial respiration, DNA repair, oxidative phosphorylation, and iron metabolism [31,32]. IREB2 is responsible for regulating iron metabolism and maintaining iron homeostasis [33]. Recent studies have shown that SARS-CoV-2 infection reduces cellular resistance to oxidative stress, promoting cellular ferroptosis [34,35]. Based on these findings, we postulated that IBV infection escalates IREB2 expression (Figure 7), potentially contributing to cellular ferroptosis [36]. The interaction between IBV Nsp2 and these host proteins suggests that Nsp2 may modulate pathways related to mitochondrial biogenesis, such as mitochondrial metabolism, iron homeostasis, and apoptosis, thereby influencing viral replication and infection.

The host proteins ATP1B1, ATP1B3, and ABCB1 are essential for maintaining ionic homeostasis (Figure 6). ATP1B1 and ATP1B3, members of the P-type ATPase superfamily, are essential for maintaining sodium and potassium ion gradients across plasma membranes [37]. Research has indicated that the interaction between the UL136 protein and ATP1B1 plays an important role in regulating cellular osmotic pressure and intracellular ion homeostasis during human cytomegalovirus (HCMV) infection [38]. ABCB1, a transporter protein driven by ATP, is responsible for transporting various hydrophobic amphiphilic compounds, including therapeutic drugs, peptides, and lipids, and significantly contributes to protecting tissues from toxic substances [39,40]. The interaction of IBV Nsp2 with these host proteins suggested that Nsp2 potentially contributes to altering the host cell environment during viral infection.

Previous studies have demonstrated the involvement of Nsp2 proteins from PEDV, SARS-CoV-1, SARS-CoV-2, and MERS-CoV in regulating innate immunity [12,41,42]. Drawing insights from the crystal structure of the IBV Nsp2 protein, scholars have postulated its potential contribution to innate immune regulation during viral infections, although the precise mechanism remains elusive [13,14]. Among the host proteins screened in this study, ATP1B1, ATP1B3, NFIA, and ITGA1 have been associated with innate immune regulation [43,44,45,46]. We suggested that the IBV Nsp2 protein might regulate host innate immunity by interacting with these host proteins, consequently impacting viral replication and proliferation.

DNAJA1, a member of the HSP40 family of heat shock proteins, functions as a molecular chaperone facilitating protein folding, stabilization, and degradation within cells [47]. Previous studies have highlighted its role in regulating viral proliferation through interaction with Japanese encephalitis virus (JEV) and influenza A virus (IAV) [48,49]. Additionally, through a high-throughput method based on random cellular gene inactivation, DNAJA1 was found to be a regulator of human immunodeficiency virus type 1 (HIV-1) replication [50]. This suggests the role of DNAJA1 in modulating multiple viral infections through diverse mechanisms. Despite this, the impact of DNAJA1 on IBV replication remains unexplored. Our study revealed a significant upregulation of DNAJA1 transcript levels in IBV-infected CEK cells and further demonstrated a positive regulatory effect of DNAJA1 on IBV replication (Figure 7). This regulation could be attributed to the Nsp2-DNAJA1 interaction, which potentially enhances viral RNA replication [51], prevents host cell apoptosis [30], inhibits host antiviral responses [52], or operates through undiscovered mechanisms. The mechanisms underlying Nsp2-DNAJA1 action in IBV infection will be further elucidated in our forthcoming research endeavors.

## 5. Conclusions

This study successfully identified 10 host proteins that interact with the IBV Nsp2 protein through yeast two-hybrid and molecular docking simulations. The functions of these host proteins were predicted through GO and KEGG analyses, and protein-protein interaction networks were constructed. These findings will improve our understanding of IBV pathogenesis and the role of Nsp2 proteins in these processes. Furthermore, the study unveiled the interaction between Nsp2 and DNAJA1, emphasizing the positive regulatory impact of DNAJA1 on viral replication. This discovery provides valuable insights for the development of novel therapeutic strategies and preventive strategies.

## Figures and Tables

**Table 1:** Primer sequences used for PCR amplification.

Primer	Sequence	Size (bp)
Nsp2-F	5′-ATGGCTTCAAGCCTAAAACAGG-3′	2025
Nsp2-R	5′-TTAACCCGCTTTGCAAATAACAT-3′	
T7-F	5′-TAATACGACTCACTATAGGGC-3′	200–2000
AD-R	5′-GTGAACTTGCGGGGTTTTTC-3′	
ATP1B3-F	5′-ATGAGCAAGGAGACGAAGAAG-3′	846
ATP1B3-R	5′-TCACTATTCAGTCATCTCAACTTTGAAGGC-3′	
DNAJA1-F	5′-ATGGTGAAGGAGACCACGTACTAC-3′	1194
DNAJA1-R	5′-TCATGATGTCTGACATTGAACACCT-3′	
ISCA1-F	5′-ATGGCATCGTCGGTGGTG-3′	388
ISCA1-R	5′-TAATGTTAAAGCTTTCTCCACA-3′	

**Table 2:** The primer sequences in qRT-PCR.

Gene	Sequence	Size (bp)
COX3-F	5′-TGACCAATCTTCGGCGCA-3′	211
COX3-R	5′-AAAGGATTATTCCGTATCGTAGGC-3′	
NFIA-F	5′-ATGTATTCTCCGCTCTGTCTC-3′	180
NFIA-R	5′-CAGTTCGTCCTTCACGGCTCT-3′	
ITGA1-F	5′-GAAAATGAGGAAGGAAAATGGGT-3′	148
ITGA1-R	5′-GCACTGAAGTAGCGTCTGGTAAAT-3′	
COX1-F	5′-CGTAGAAGCTGGGGCCGG-3′	230
COX1-R	5′-GGATGGCAGTAATGAGGACGGA-3′	
ATP1B1-F	5′-CTGCAAGTTCAAACGTGAGTGG-3′	195
ATP1B1-R	5′-CAGTGGACAGGGATGAGATAGGG-3′	
ABCB1-F	5′-GAAATACATATGAGATCGCTA-3′	101
ABCB1-R	5′-CGGGCTGACCATTTGAGGCT-3′	
ISCA1-F	5′-GGCATCGTCGGTGGTGCGGGC-3′	137
ISCA1-R	5′-CTACATGCTCAGGCTGGTCTT-3′	
DNAJA1-F	5′-TGGCACTGAAGTACCACCCC-3′	85
DNAJA1-R	5′-TCGGACAGCACCTCATACGC-3′	
ATP1B3-F	5′-ATGAGCAAGGAGACGAAGAAGC-3′	154
ATP1B3-R	5′-CCGCGAGGAAGCCATAAAAT-3′	
IREB2-F	5′-ATACAGAACGCCCCGAACCCT-3′	204
IREB2-R	5′-AAGGTGGAAAGGGCAGAGGA-3′	
β-actin-F	5′-CTGTGCCCATCTATGAAGGCTA-3′	139
β-actin-R	5′-ATTTCTCTCTCGGCTGTGGTG-3′	

**Table 3:** Potential host proteins that interact with the Nsp2 protein.

Gene	NCBI Accession	Function
COX1	QFK69789.1	Component regarding the cytochrome c oxidase. The last enzyme in the mitochondrial electron transport chain drives oxidative phosphorylation.
COX3	QFK69793.1	
NFIA	XM_038183087.1	DNA-binding transcription factor activity.
ITGA1	NM_205069.1	Involved in the anchorage-dependent, negative regulation of EGF-stimulated cell growth.
ATP1B1	NM_205520.4	Catalyzes the hydrolysis of ATP coupled with the exchange of Na^+^ and K^+^ ions across the plasma membrane.
ATP1B3	NM_205535.1	
ABCB1	XM_025147038.1	Translocate drugs and phospholipids across the membrane.
ISCA1	NM_001271936.1	Involved in the maturation of mitochondrial 4Fe-4S proteins and functioning late in the iron-sulfur cluster assembly.
DNAJA1	NM_001012945	Plays a role in protein transport into mitochondria.
IREB2	NM_001031454.1	Binding to the IRE element in ferritin results in its repression.

## Data Availability

The data are available upon request from the corresponding authors.

## Supplementary Materials

The following supporting information can be downloaded at: https://www.mdpi.com/article/10.3390/vetsci11110531/s1, Table S1: Proteins that interact with Nsp2 by yeast two-hybrid screening; Figure S1: Electrophoretic analysis of total RNA extracted from chicken kidney tissue; Figure S2: Identification of the inserted DNAs from the cDNA library; Figure S3: PCR products from positive clones of the cDNA library; Figure S4: Co-IP analysis to identify Nsp2 interaction with host proteins; Figure S5: Effect of DNAJA1 overexpression on IBV replication; Figure S6: Effect of interfering with DNAJA1 expression on IBV replication.
