# Supplementary material for: Interaction Network Characterization of Infectious Bronchitis Virus Nsp2 with Host Proteins
Source: Vet Sci. 2024 Oct 31;11(11):531. doi: 10.3390/vetsci11110531 (PMC11598884; doi:10.3390/vetsci11110531)
Supplement: Supplementary file 1 [file vetsci-11-00531-s001.zip › Table S1.pdf]

Table S1. Proteins that interact with Nsp2 by yeast two-hybrid screening

No.	Protein name	NCBI accession	Number of clones	ORF (bp)
1	ATP1B1	NM_205520.4	51	918
2	ISCA1	NM_001271936.1	4	390
3	ATP1B3	NM_205535.1	3	843
4	COX3	QFK69793.1	2	783
5	FAM96B	XM_414150.6	2	486
6	NET1	XM_015284331.2	2	1665
7	PECAM1	XM_040686404.1	2	2037
8	ABCB1	XM_025147038.1	1	3867
9	AKIP1	XR_005850716.1	1	1101
10	COL4A4	XM_040679288	1	5769
11	COX1	QFK69789.1	1	1548
12	DNAJA1	NM_001012945	1	1194
13	IREB2	NM_001031454.1	1	2898
14	ITGA1	NM_205069.1	1	3516
15	LEO1	NM_001292086.3	1	1974
16	LOXL1	NM_005576.4	1	1725
17	ND1	QFK69774	1	972
18	NFIA	XM_038183087.1	1	1368
19	PCNA	NM_204170.2	1	789
20	PSMB1	NM_001007905.2	1	714
21	RARRES2	NM_001277477	1	489
22	RPL12	NM_001277679.1	1	498
23	SELENBP1	NM_001277810.1	1	1416
24	SORBS2	XM_040671297	1	2733
25	THEM4	XM_015280018.2	1	570
