# Supplementary figures and images for: Interaction Network Characterization of Infectious Bronchitis Virus Nsp2 with Host Proteins
Source: Vet Sci. 2024 Oct 31;11(11):531. doi: 10.3390/vetsci11110531 (PMC11598884; doi:10.3390/vetsci11110531)

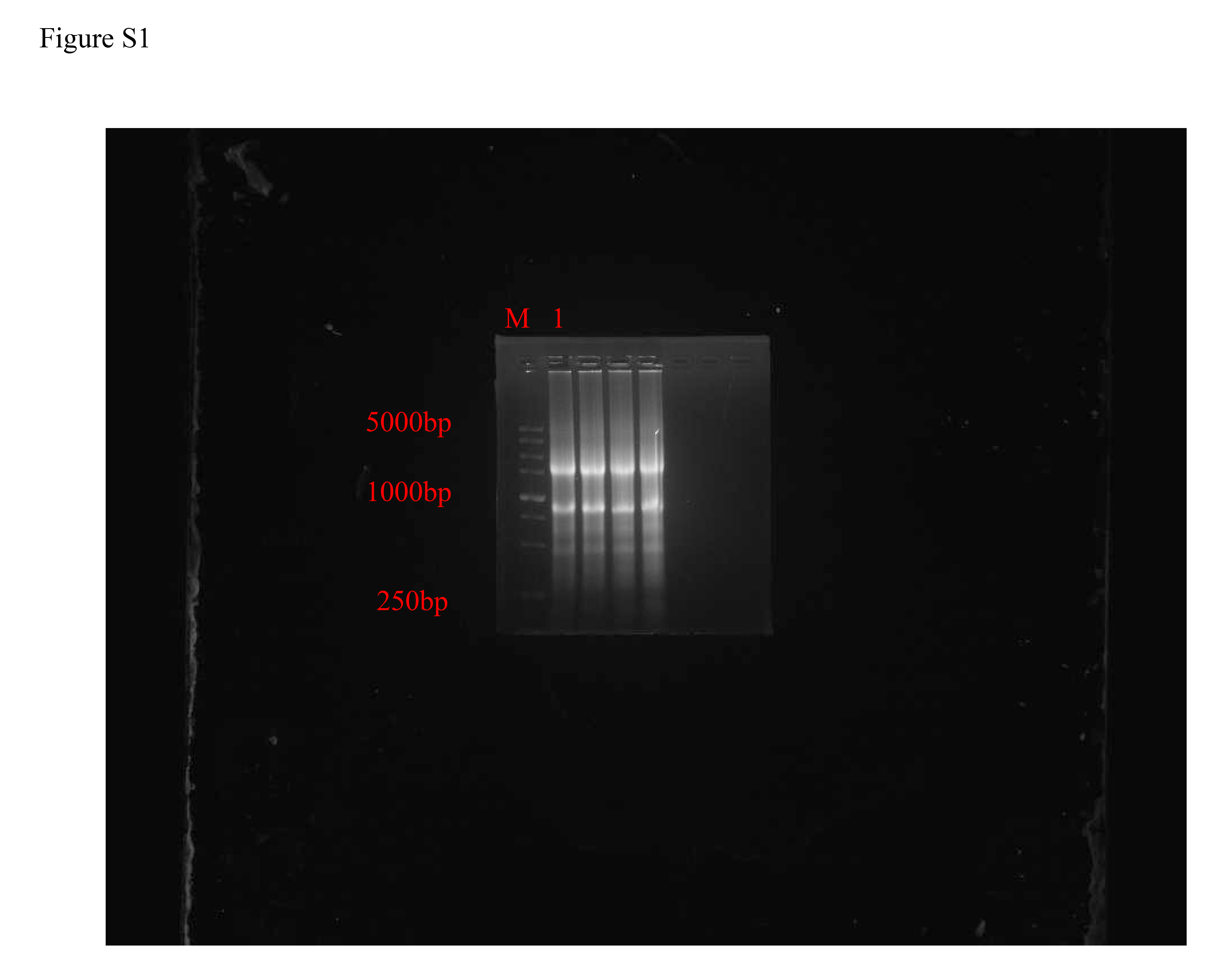

Supplement: Supplementary file 1 [file vetsci-11-00531-s001.zip › Figure S1/Figure S1-.Tif]

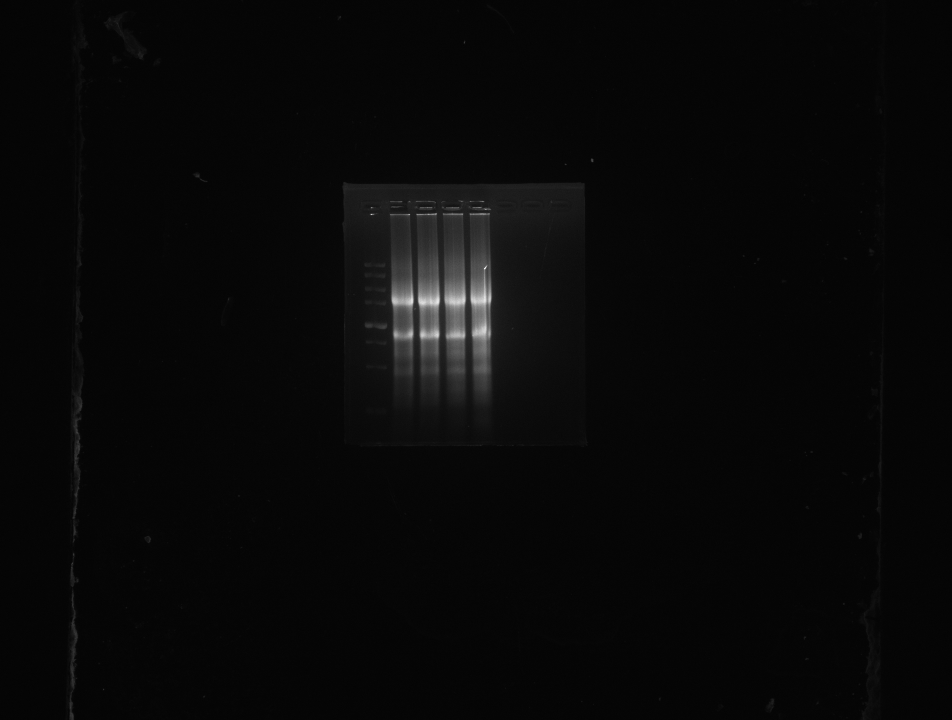

Supplement: Supplementary file 1 [file vetsci-11-00531-s001.zip › Figure S1/Figure S1.Tif]

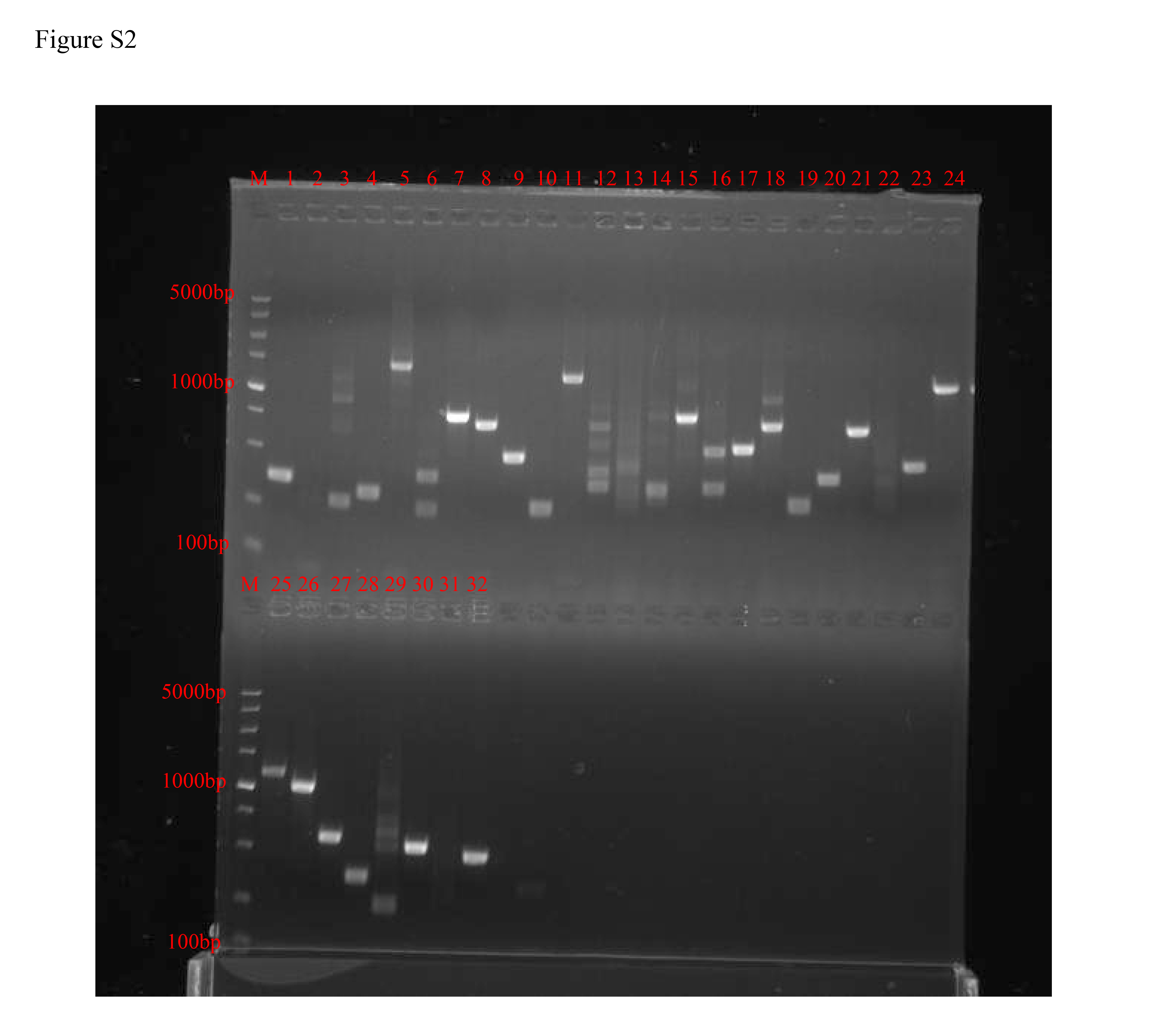

Supplement: Supplementary file 1 [file vetsci-11-00531-s001.zip › Figure S2/Figure S2-.Tif]

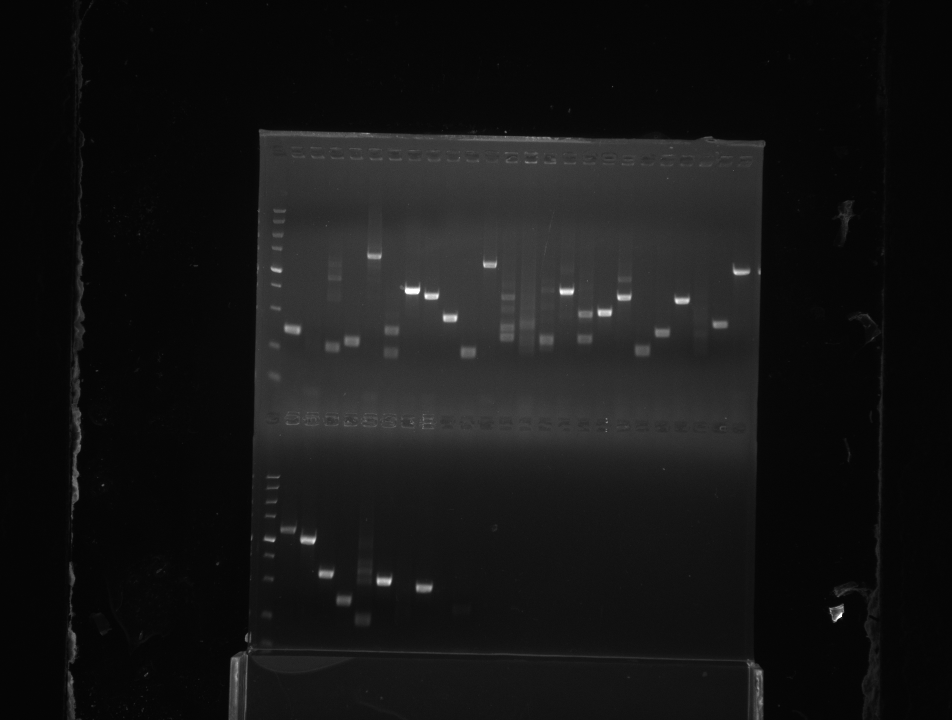

Supplement: Supplementary file 1 [file vetsci-11-00531-s001.zip › Figure S2/Figure S2-M,1-24;M,25-36.Tif]

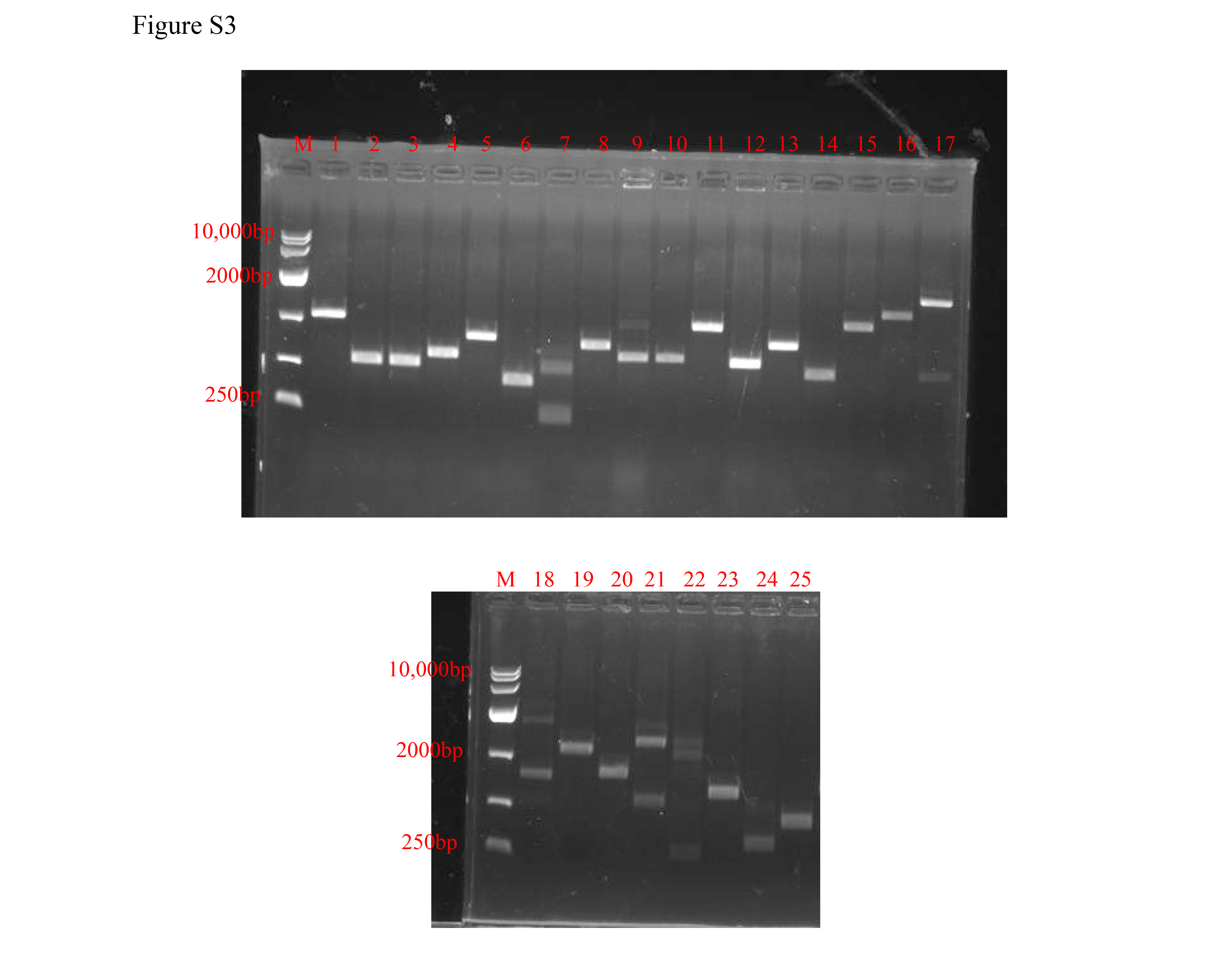

Supplement: Supplementary file 1 [file vetsci-11-00531-s001.zip › Figure S3/Figure S3-.tif]

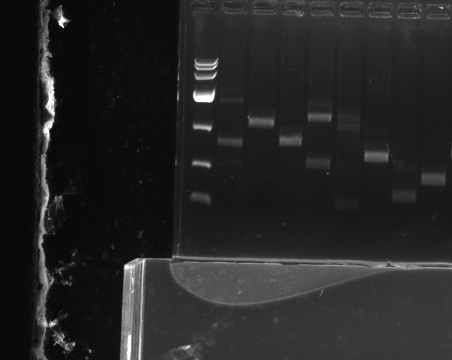

Supplement: Supplementary file 1 [file vetsci-11-00531-s001.zip › Figure S3/Figure S3; M, 18-25.tif]

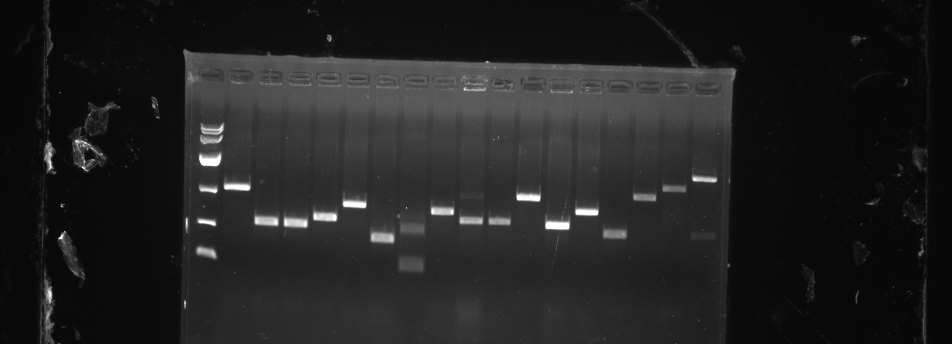

Supplement: Supplementary file 1 [file vetsci-11-00531-s001.zip › Figure S3/Figure S3; M,1-17.tif]

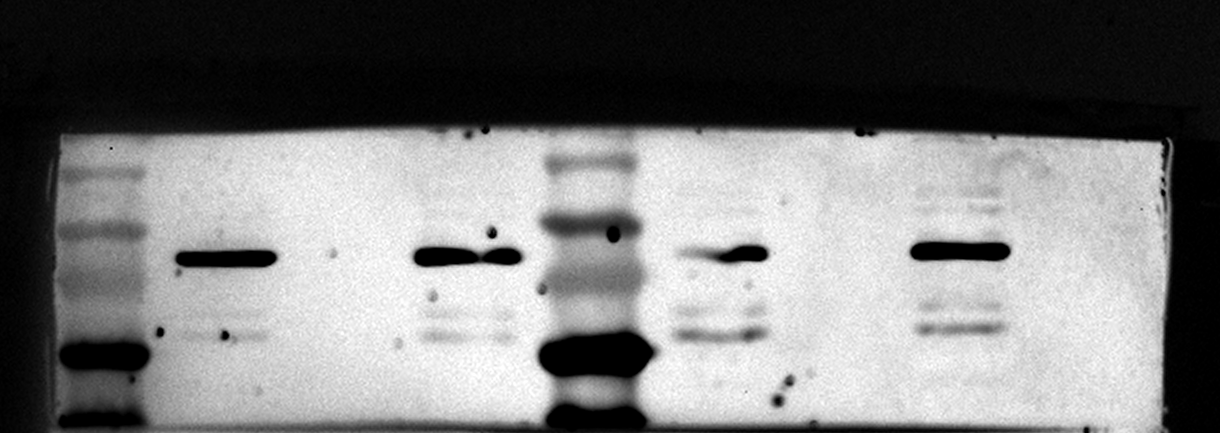

Supplement: Supplementary file 1 [file vetsci-11-00531-s001.zip › Figure S4/Figure S4, Nsp2+ATP1B3, IP-Flag, αFlag; Figure S4, Nsp2+DNAJA1, IP-Flag, αFlag.tif]

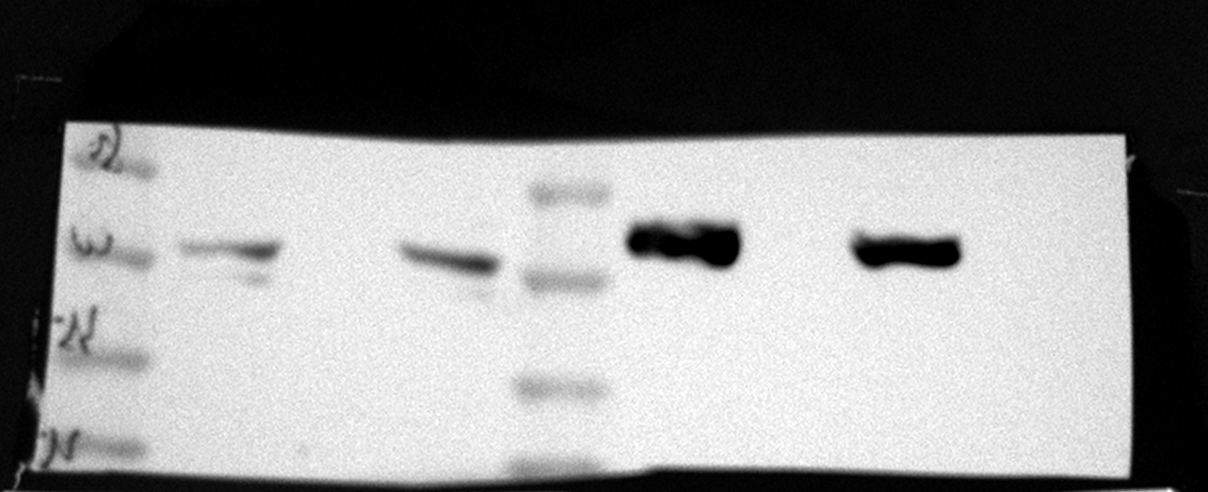

Supplement: Supplementary file 1 [file vetsci-11-00531-s001.zip › Figure S4/Figure S4, Nsp2+ATP1B3, IP-Flag, αMyc; Figure S4, Nsp2+DNAJA1, IP-Flag, αMyc.tif]

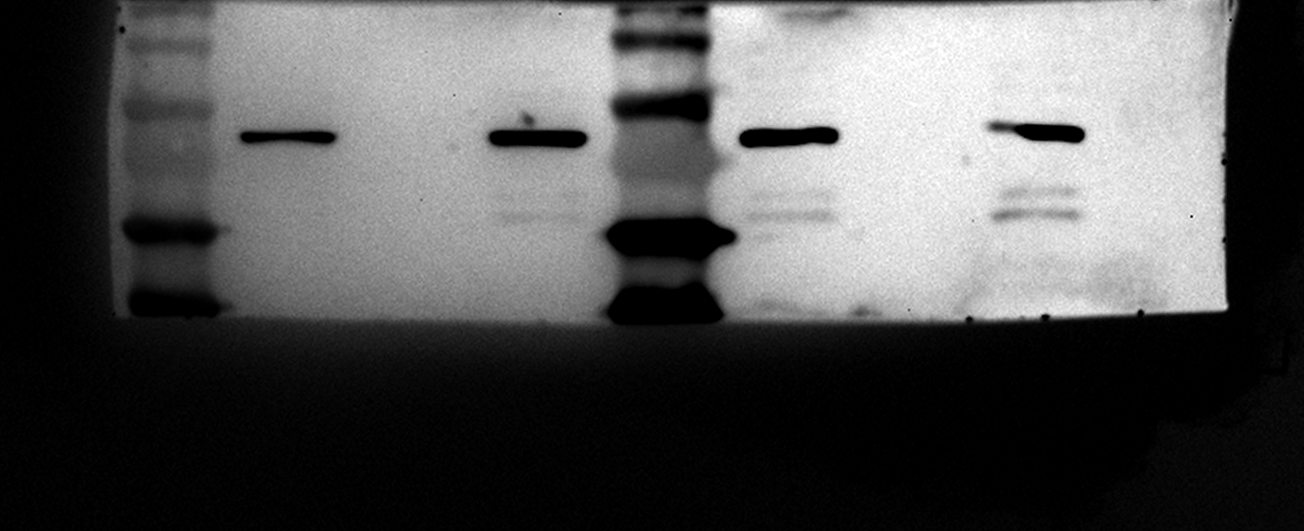

Supplement: Supplementary file 1 [file vetsci-11-00531-s001.zip › Figure S4/Figure S4, Nsp2+ATP1B3, IP-Myc, αFlag; Figure S4, Nsp2+DNAJA1, IP-Myc, αFlag.tif]

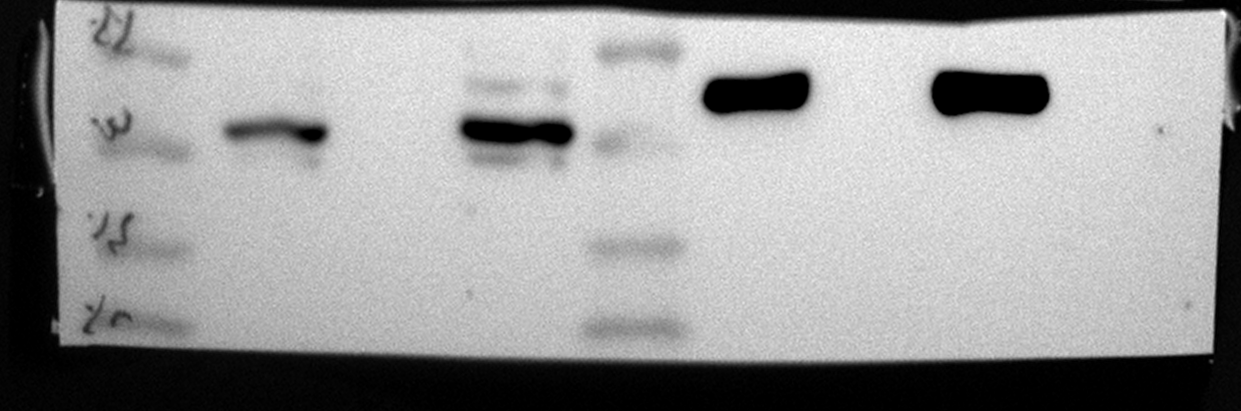

Supplement: Supplementary file 1 [file vetsci-11-00531-s001.zip › Figure S4/Figure S4, Nsp2+ATP1B3, IP-Myc, αMyc; Figure S4, Nsp2+DNAJA1, IP-Myc, αMyc.tif]

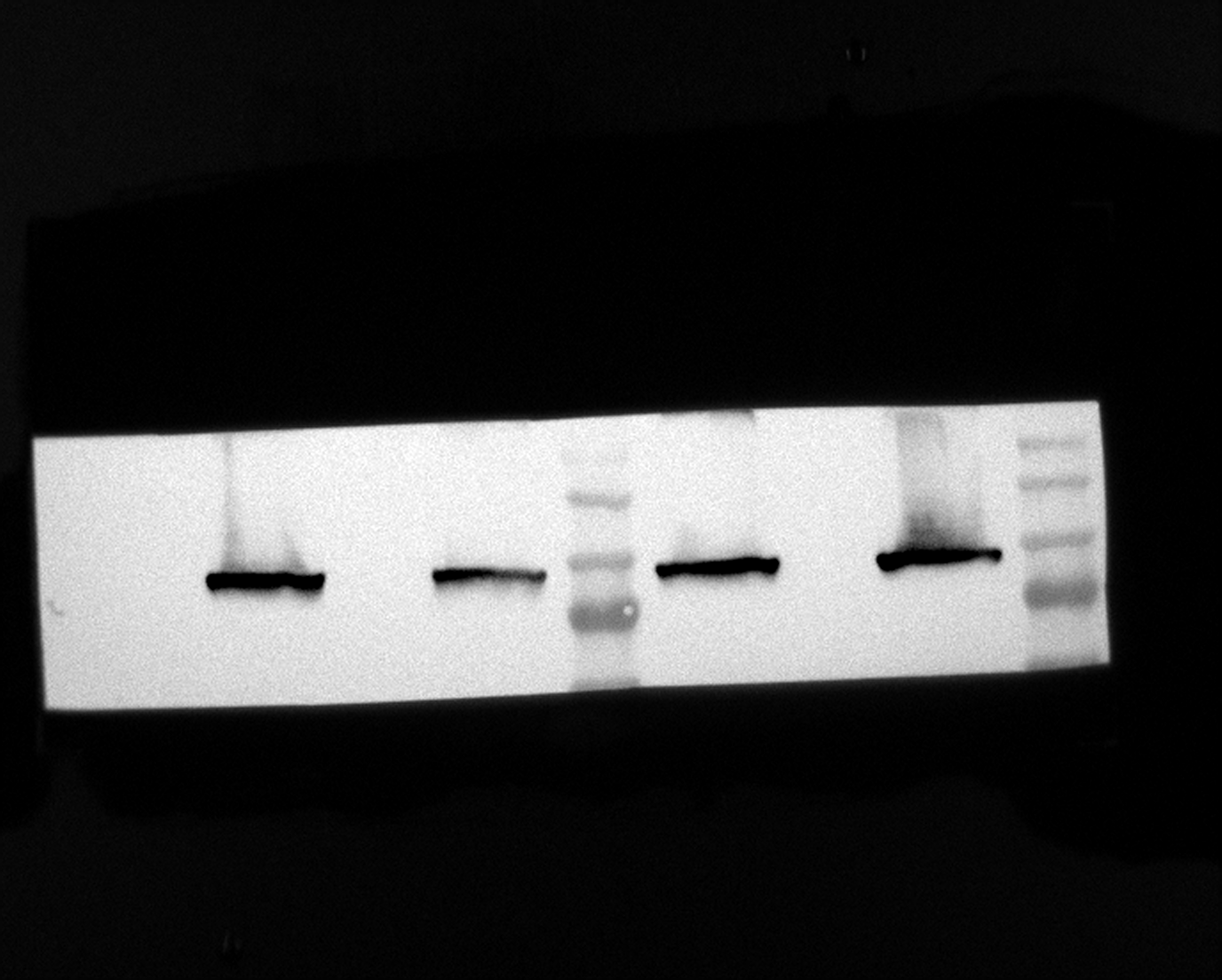

Supplement: Supplementary file 1 [file vetsci-11-00531-s001.zip › Figure S4/Figure S4, Nsp2+ISCA1, IP-Myc, αFlag; Figure S4, Nsp2+ISCA1, IP-Flag, αFlag.tif]

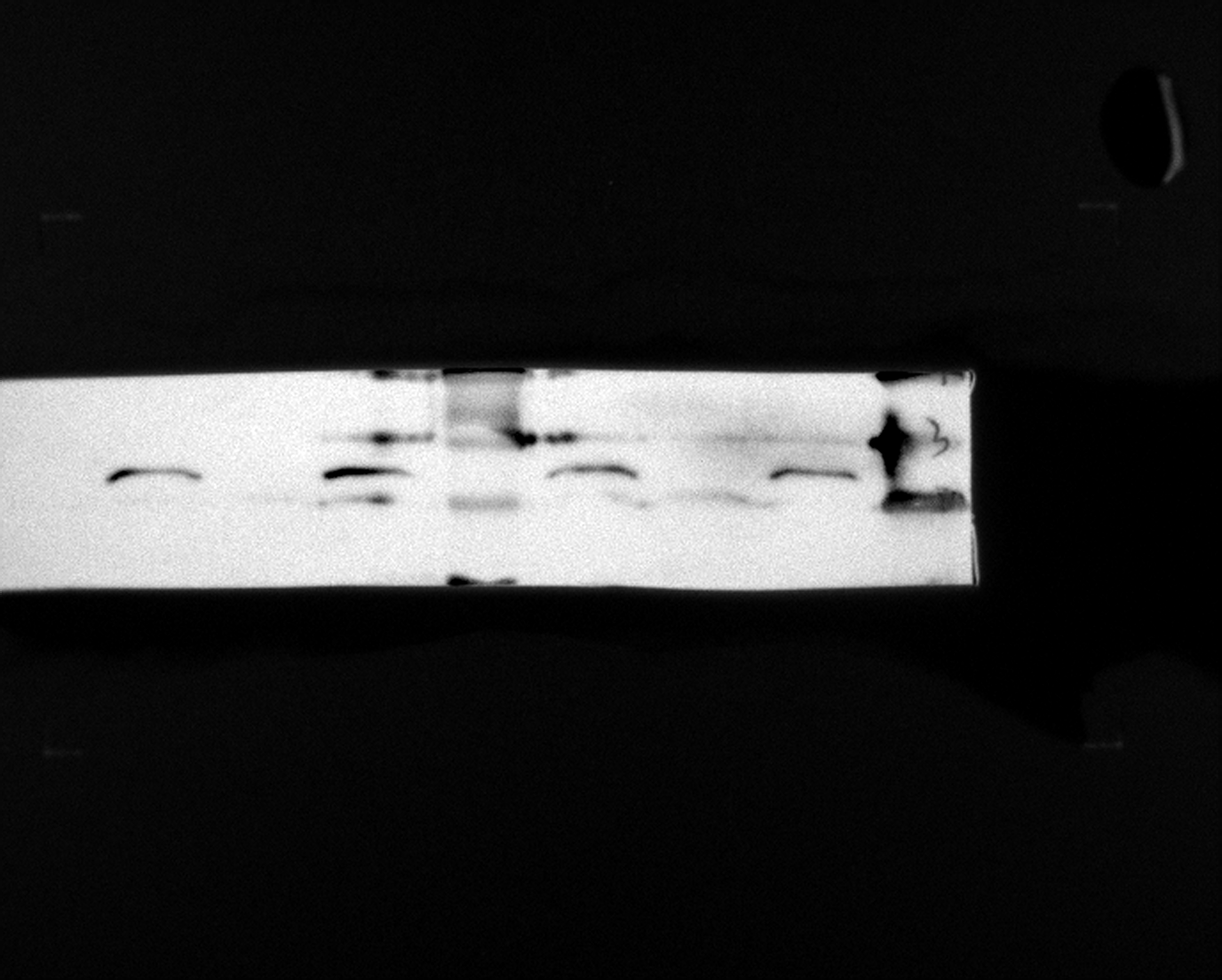

Supplement: Supplementary file 1 [file vetsci-11-00531-s001.zip › Figure S4/Figure S4, Nsp2+ISCA1, IP-Myc, αMyc; Figure S4, Nsp2+ISCA1, IP-Flag, αMyc.tif]

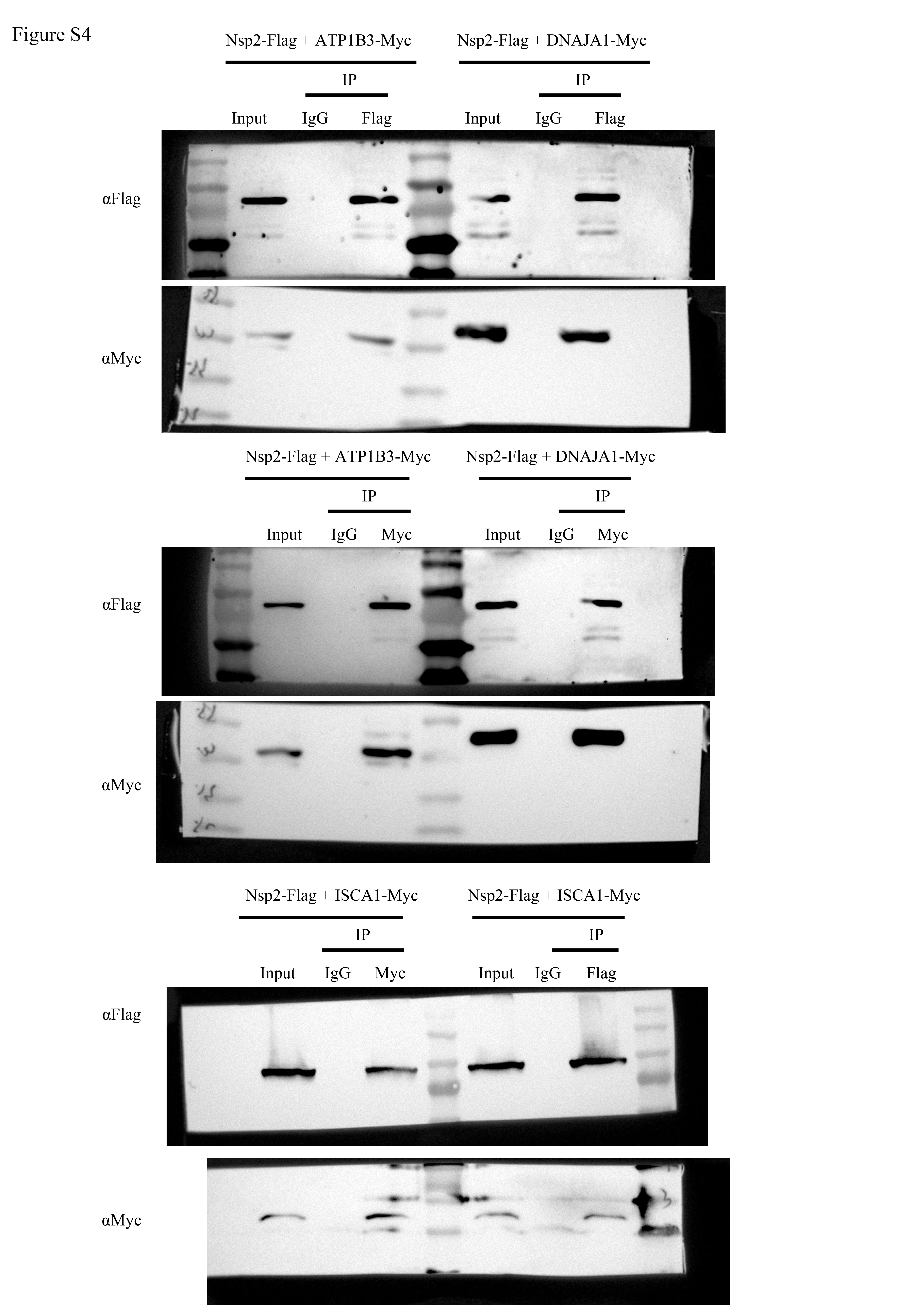

Supplement: Supplementary file 1 [file vetsci-11-00531-s001.zip › Figure S4/Figure S4-.tif]

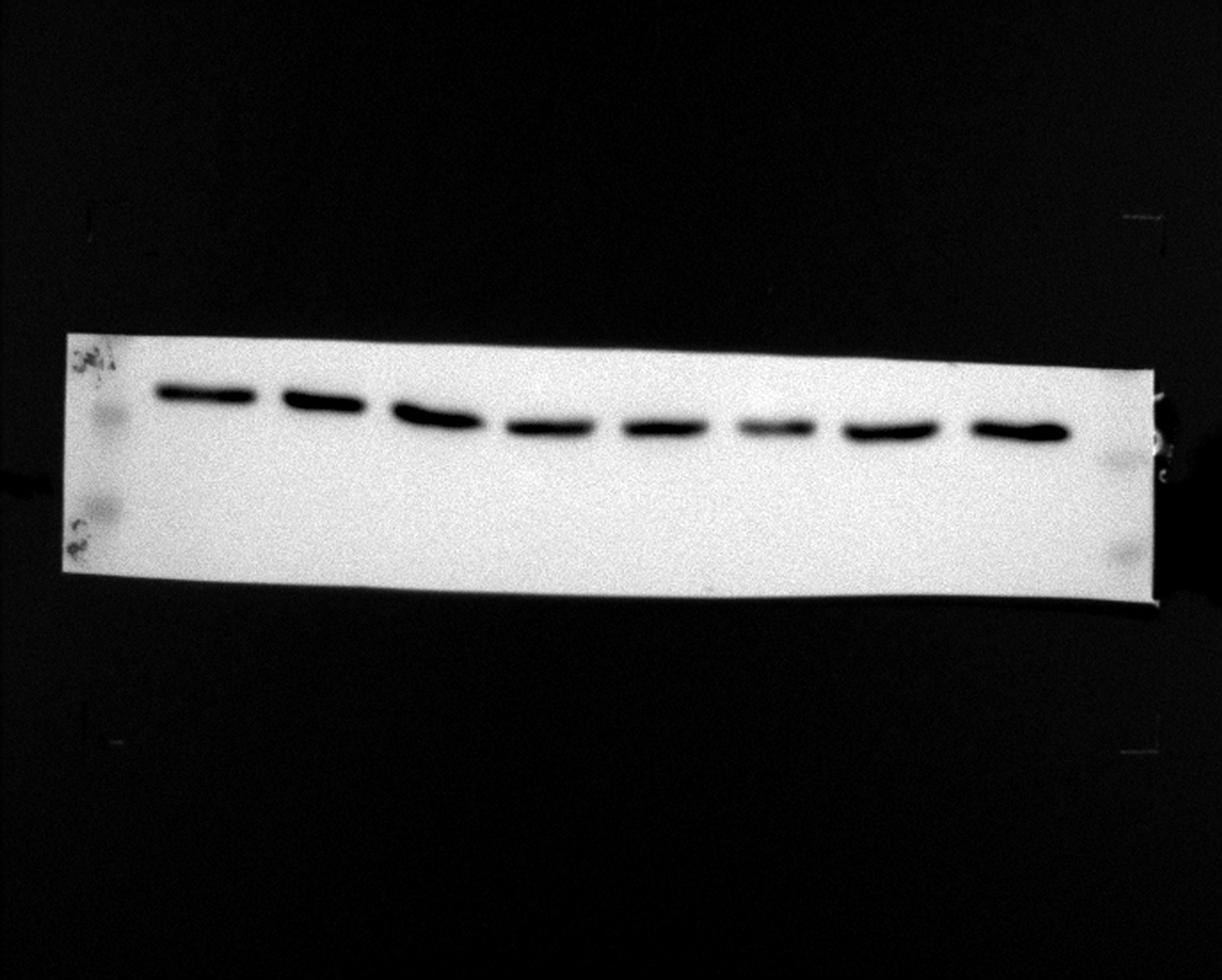

Supplement: Supplementary file 1 [file vetsci-11-00531-s001.zip › Figure S5/Figure S5, αGAPDH-M,Vector 12h,DNAJA1 12h,Vector 24h,DNAJA1 24h, Vector 36h, DNAJA1 36h, Vector 48h, DNAJA1 48h.tif]

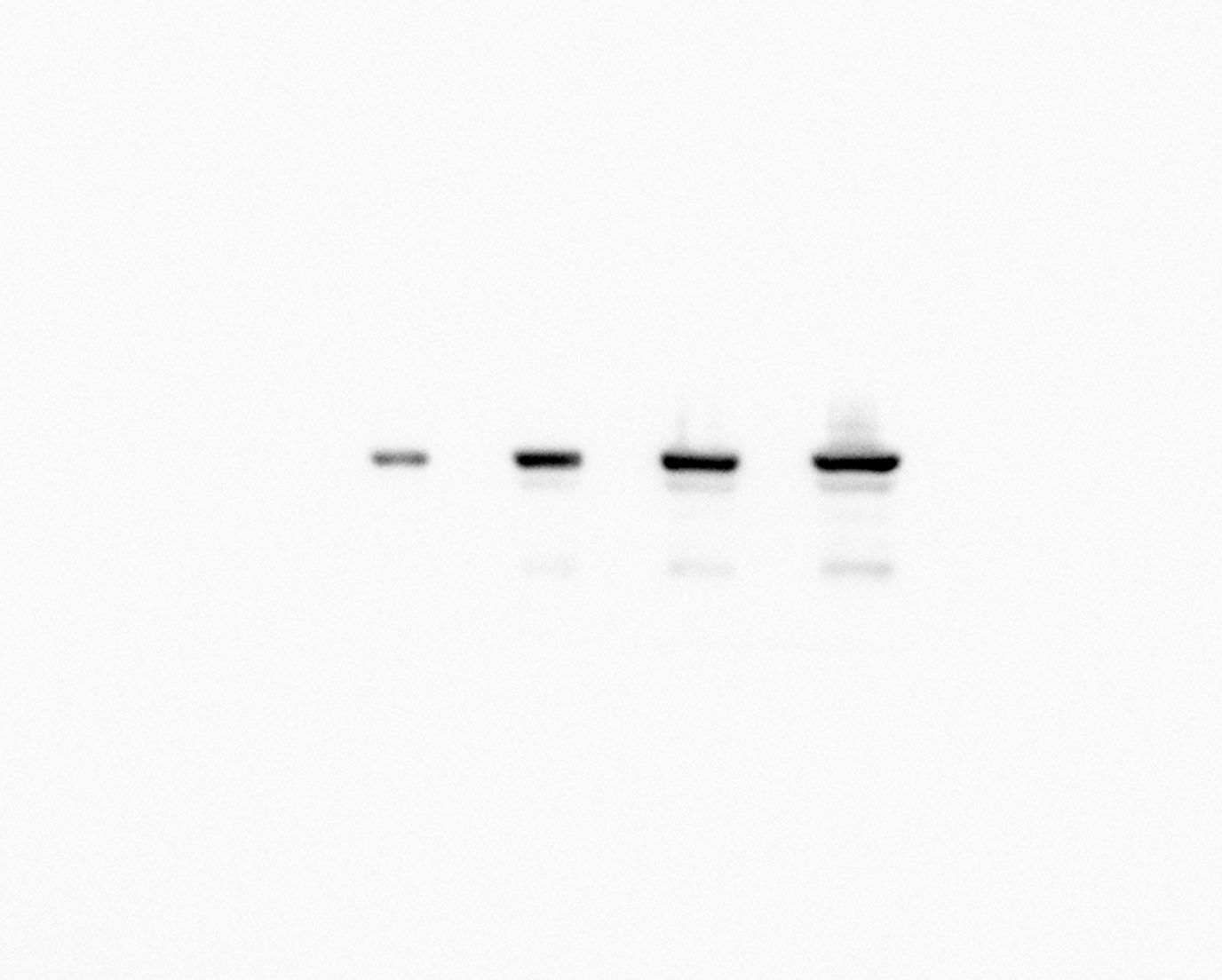

Supplement: Supplementary file 1 [file vetsci-11-00531-s001.zip › Figure S5/Figure S5, αMyc-Vector12h,DNAJA1 12h,Vector 24h,DNAJA1 24h, Vector 36h, DNAJA1 36h, Vector 48h, DNAJA1 48h .tif]

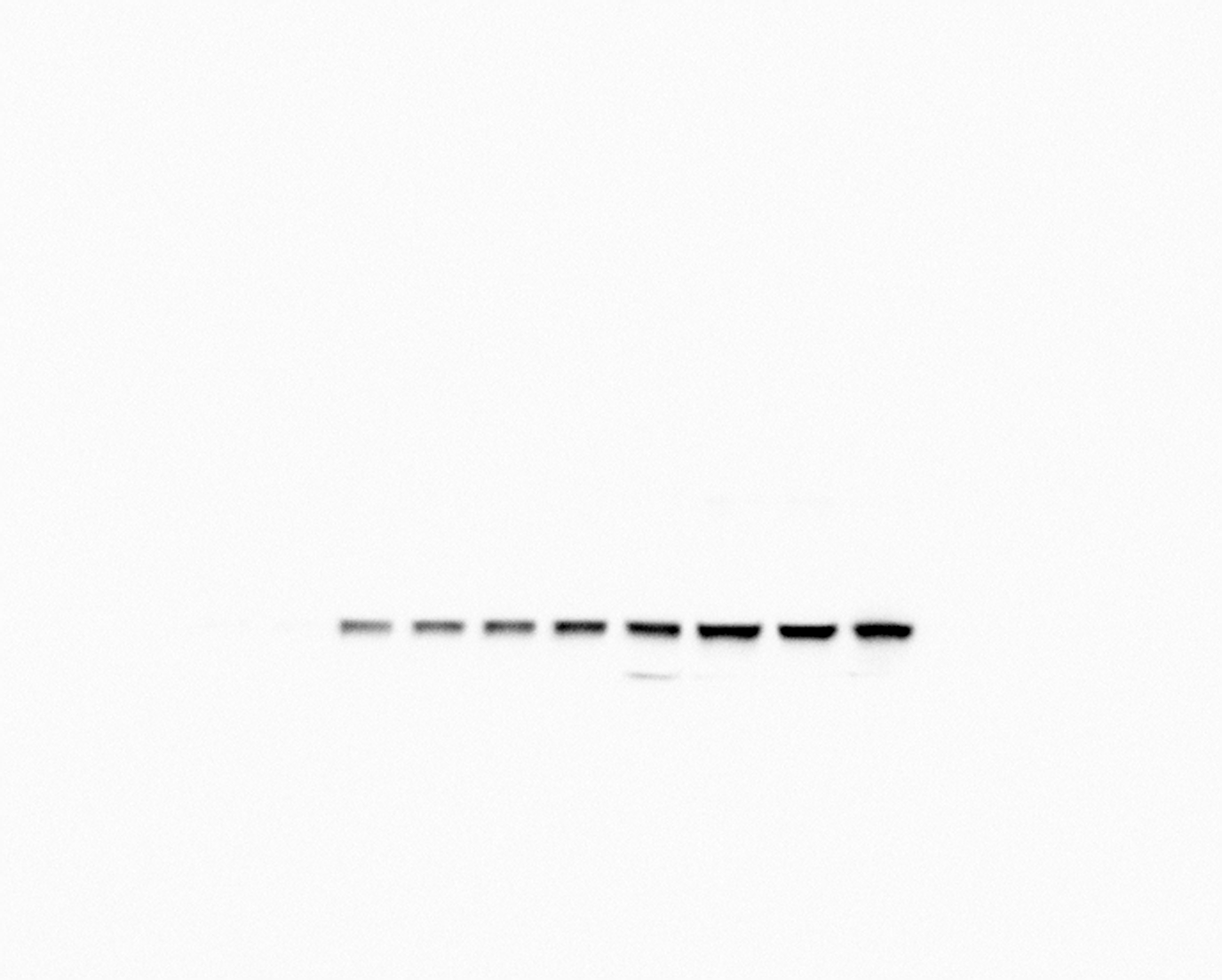

Supplement: Supplementary file 1 [file vetsci-11-00531-s001.zip › Figure S5/Figure S5, αN-Vector 12h,DNAJA112h,Vector 24h,DNAJA1 24h, Vector 36h, DNAJA1 36h, Vector 48h, DNAJA1 48h.tif]

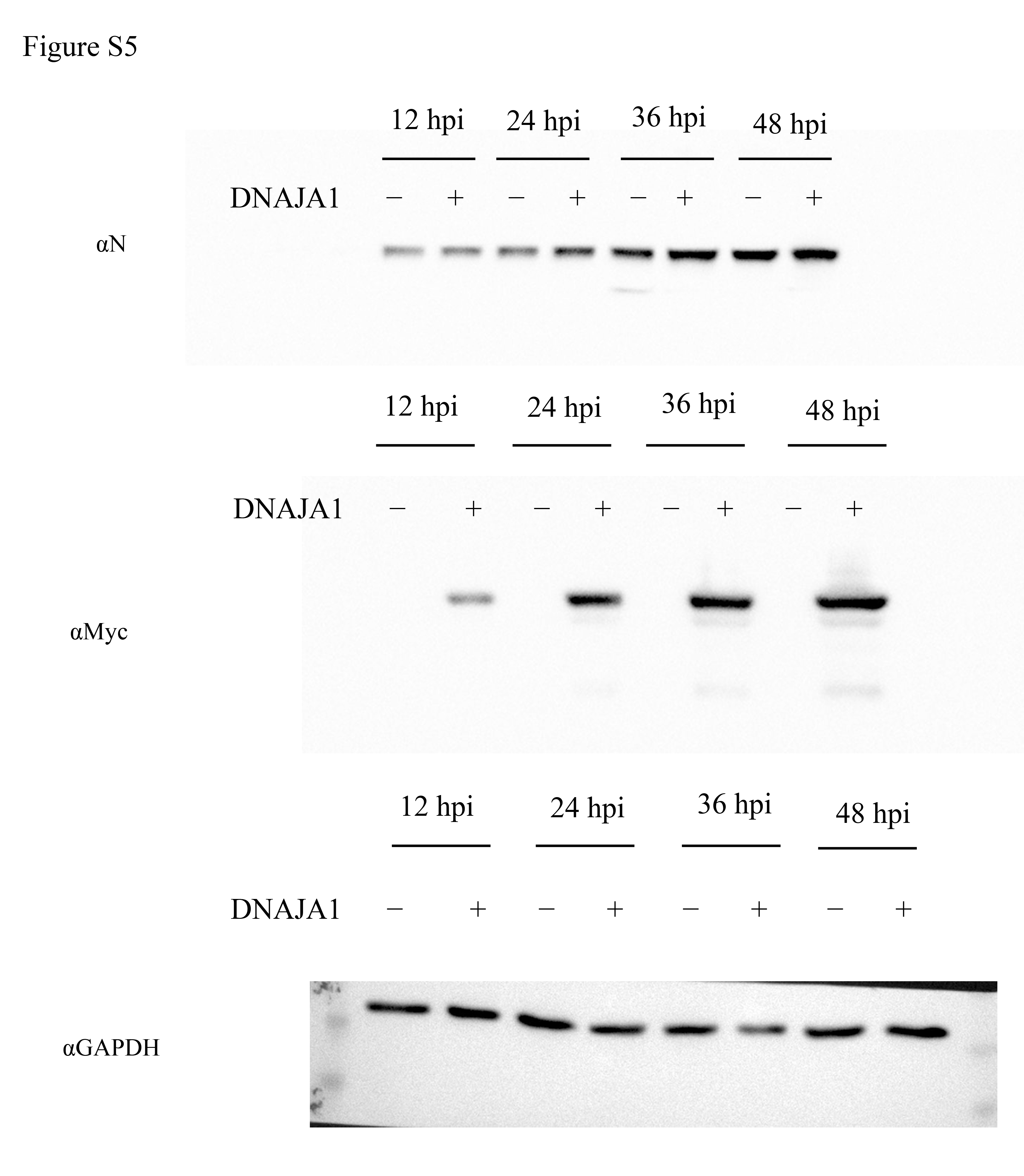

Supplement: Supplementary file 1 [file vetsci-11-00531-s001.zip › Figure S5/Figure S5-.tif]

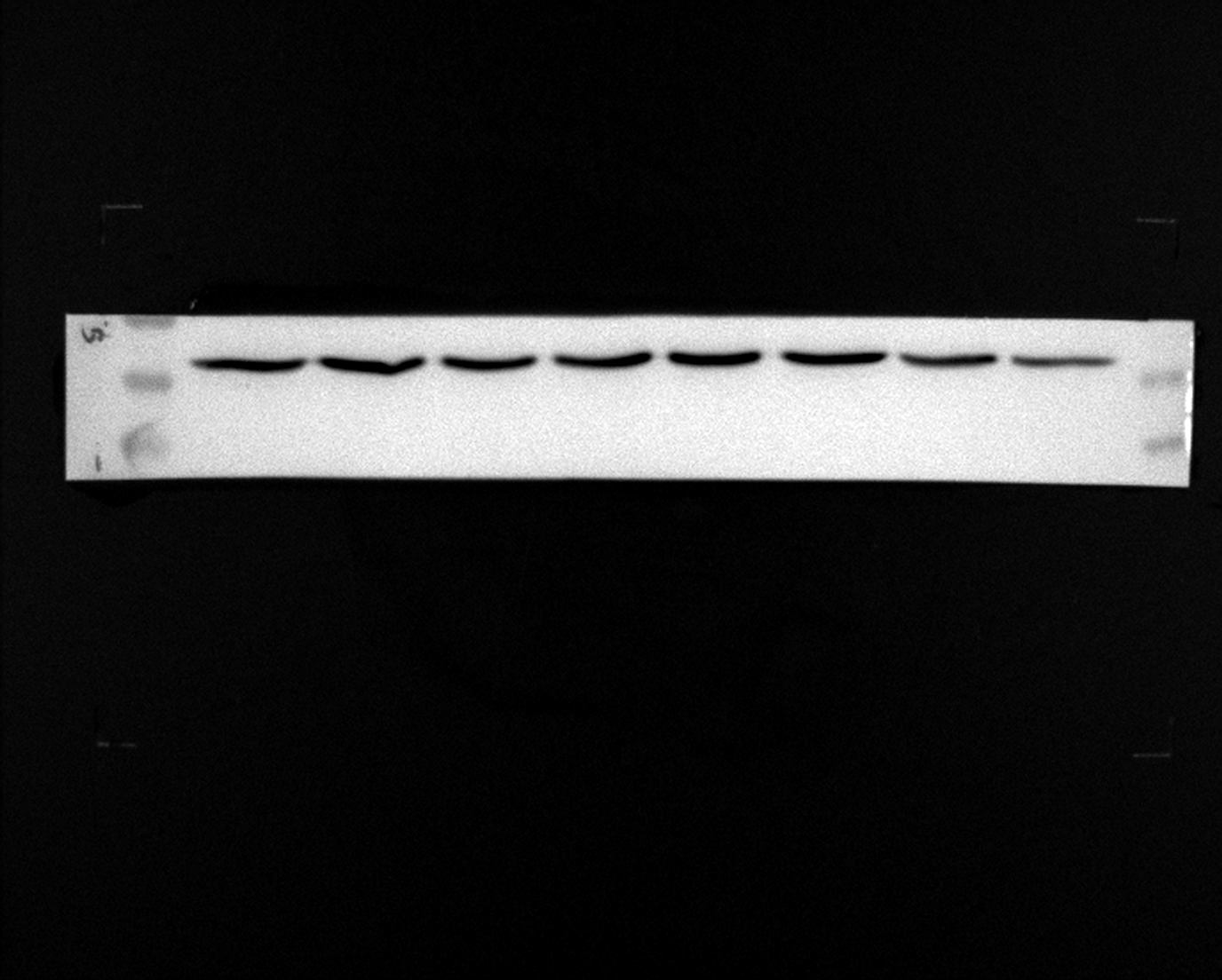

Supplement: Supplementary file 1 [file vetsci-11-00531-s001.zip › Figure S6/Figure S6, αGAPDH-M,siNC 12h,siDNAJA1 12h,siNC 24h,siDNAJA1 24h, siNC 36h, siDNAJA1 36h, siNC 48h, siDNAJA1 48h.tif]

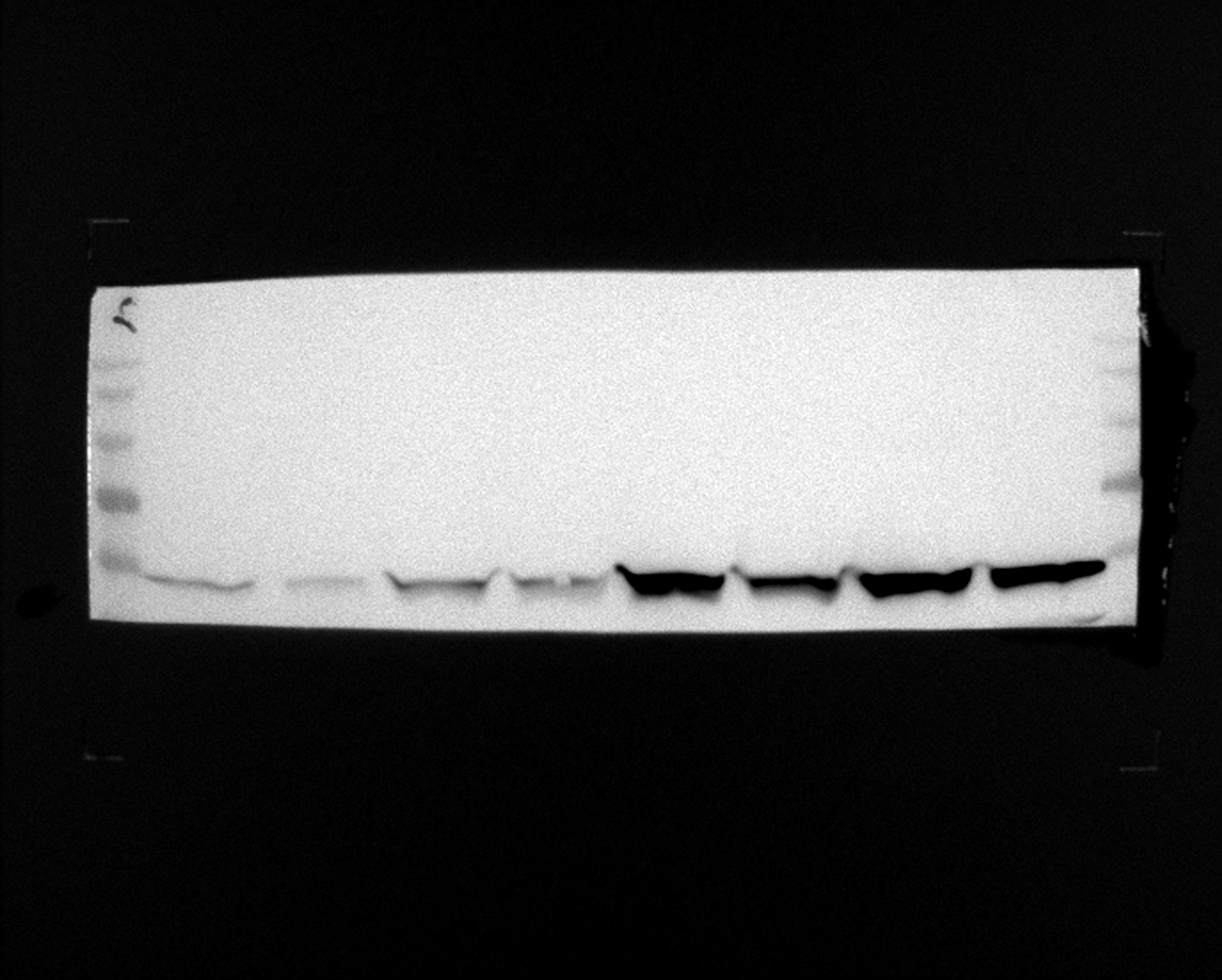

Supplement: Supplementary file 1 [file vetsci-11-00531-s001.zip › Figure S6/Figure S6, αN-M,siNC 12h,siDNAJA1 12h,siNC 24h,siDNAJA1 24h, siNC 36h, siDNAJA1 36h, siNC 48h, siDNAJA1 48h.tif]

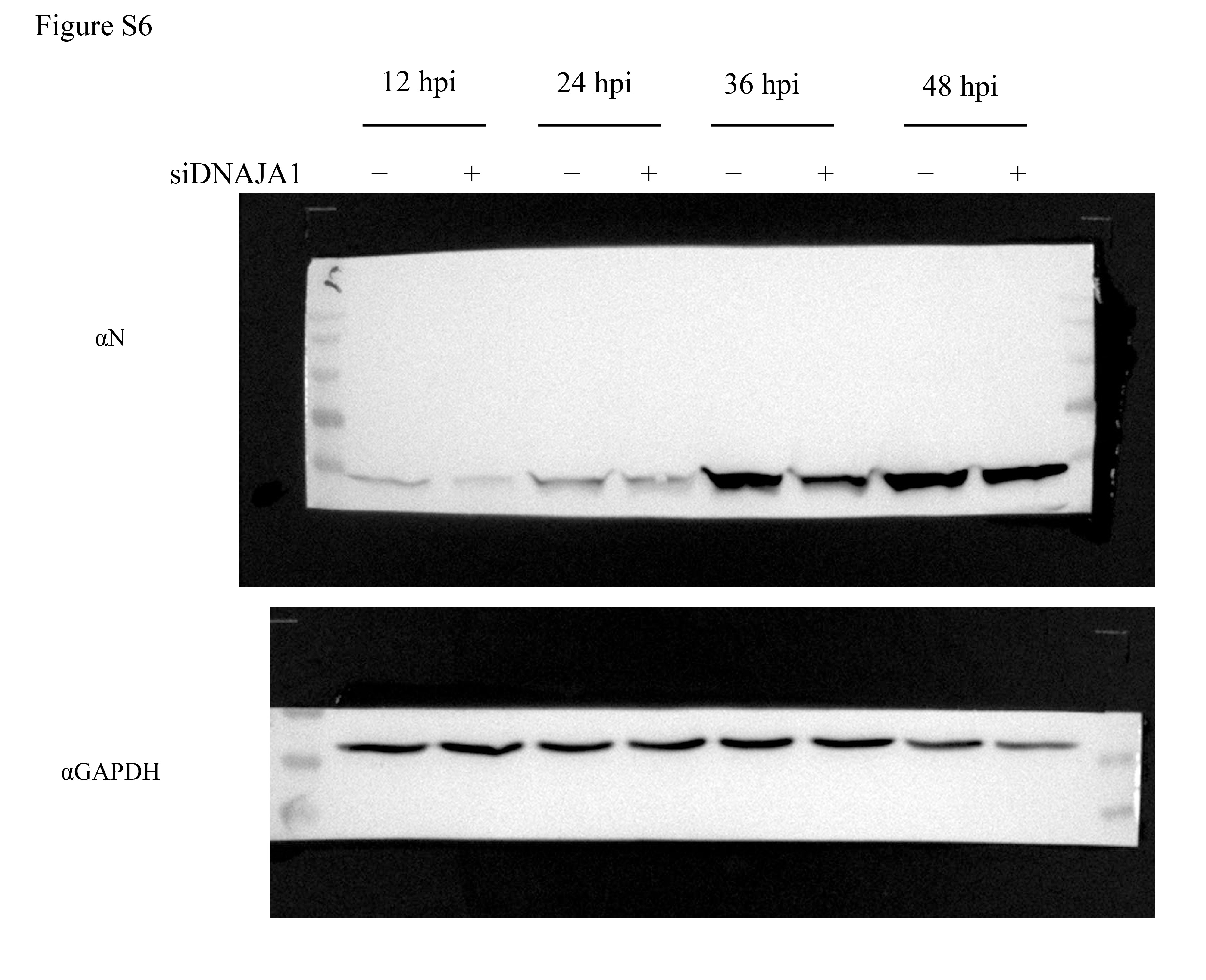

Supplement: Supplementary file 1 [file vetsci-11-00531-s001.zip › Figure S6/Figure S6-.tif]
